# MicroRNA-regulated pathways of flow-stimulated angiogenesis and vascular remodeling in vivo

**DOI:** 10.1186/s12967-019-1767-9

**Published:** 2019-01-11

**Authors:** Dominic Henn, Masood Abu-Halima, Dominik Wermke, Florian Falkner, Benjamin Thomas, Christoph Köpple, Nicole Ludwig, Matthias Schulte, Marc A. Brockmann, Yoo-Jin Kim, Justin M. Sacks, Ulrich Kneser, Andreas Keller, Eckart Meese, Volker J. Schmidt

**Affiliations:** 1 Department of Hand, Plastic and Reconstructive Surgery, University of Heidelberg, BG Trauma Center Ludwigshafen, Ludwig-Guttmann Str. 13, 67071 Ludwigshafen, Germany; 2 Institute of Human Genetics, Saarland University, Homburg-Saar, Germany; 3 Institute of Clinical Bioinformatics, Saarland University, Saarbruecken, Germany; 4 Department of Neuroradiology, University Medical Center Mainz, Mainz, Germany; 5 Institute of Pathology, Kaiserslautern, Germany; 6 Department of Plastic and Reconstructive Surgery, Johns Hopkins University School of Medicine, Baltimore, MD USA

**Keywords:** AV shunt, Shear stress, Microarray, Chemokines

## Abstract

**Background:**

Vascular shear stress promotes endothelial cell sprouting in vitro. The impact of hemodynamic forces on microRNA (miRNA) and gene expression within growing vascular networks in vivo, however, remain poorly investigated. Arteriovenous (AV) shunts are an established model for induction of neoangiogenesis in vivo and can serve as a tool for analysis of hemodynamic effects on miRNA and gene expression profiles over time.

**Methods:**

AV shunts were microsurgically created in rats and explanted on postoperative days 5, 10 and 15. Neoangiogenesis was confirmed by histologic analysis and micro-computed tomography. MiRNA and gene expression profiles were determined in tissue specimens from AV shunts by microarray analysis and quantitative real-time polymerase chain reaction and compared with sham-operated veins by bioinformatics analysis. Changes in protein expression within AV shunt endothelial cells were determined by immunohistochemistry.

**Results:**

Samples from AV shunts exhibited a strong overexpression of proangiogenic cytokines, oxygenation-associated genes (HIF1A, HMOX1), and angiopoetic growth factors. Significant inverse correlations of the expressions of miR-223-3p, miR-130b-3p, miR-19b-3p, miR-449a-5p, and miR-511-3p which were up-regulated in AV shunts, and miR-27b-3p, miR-10b-5p, let-7b-5p, and let-7c-5p, which were down-regulated in AV shunts, with their predicted interacting targets C–X–C chemokine receptor 2 (CXCR2), interleukin-1 alpha (IL1A), ephrin receptor kinase 2 (EPHA2), synaptojanin-2 binding protein (SYNJ2BP), forkhead box C1 (FOXC1) were present. CXCL2 and IL1A overexpression in AV shunt endothelium was confirmed at the protein level by immunohistochemistry.

**Conclusions:**

Our data indicate that flow-stimulated angiogenesis is determined by an upregulation of cytokines, oxygenation associated genes and miRNA-dependent regulation of FOXC1, EPHA2 and SYNJ2BP.

**Electronic supplementary material:**

The online version of this article (10.1186/s12967-019-1767-9) contains supplementary material, which is available to authorized users.

## Background

Vascular remodeling and angiogenesis play important roles in the pathophysiology of cardiovascular diseases. The development of new blood vessels is governed by an interplay of biochemical and mechanical stimuli. The pulsatile blood flow generated by the cardiac cycle exposes endothelial cells (ECs) to two mechanical forces, namely circumferential stretch acting perpendicularly, and shear stress acting tangentially to the vascular wall [1]. Elevated shear stress has been shown to promote EC migration and regeneration, as well as differentiation of embryonic stem cells into ECs [2, 3]. In vitro studies have shown that mechanical forces acting on ECs and vascular smooth muscle cells (VSMCs) are translated into biochemical signals by mechanosensory proteins [4, 5]. These promote intracellular pathways, which lead to altered gene expression profiles [6, 7] with up-regulated proangiogenic factors like vascular endothelial growth factor (VEGF) [3].

Angiogenesis, however, is a delicately regulated process, which goes far beyond EC sprouting, and requires a concerted action of ECs, VSMCs, as well as signals from the extracellular matrix (ECM) and cells like macrophages and fibroblasts chemotactically attracted to areas of neoangiogenesis [8, 9]. In vitro studies on ECs have shown that elevated shear stress induces the expression of microRNAs (miRNAs) that affect vascular remodeling and angiogenesis [10, 11]. Current knowledge of differential signaling in angiogenesis largely stems from in vitro studies by means of EC sprouting assays or co-culture systems [12]. Still, these models are limited in representing in vivo physiologic conditions. The development of new therapeutic strategies targeting angiogenesis requires more realistic model systems and a more detailed understanding of the complex in vivo environment determining angiogenic processes. Therefore, studies integrating the molecular signals from different cellular players and miRNAs which govern angiogenesis are needed.

So far, evidence is lacking on how elevated shear stress in vivo influences miRNA expression profiles within the vascular wall, and to what extent altered miRNA signatures influence the expression levels of pro- and anti-angiogenic genes. This knowledge may soon gain clinical value as RNA-based therapeutics are an emerging field in cardiovascular pharmacology, and studies in small and large animal models of cardiovascular diseases have yielded promising results [13–15]. MiRNA-mediated silencing of anti-angiogenic genes by means of synthetic RNA mimics appears to be a promising approach for the promotion of local neoangiogenesis in ischemic myocardium [16]. Conversely, miRNA-mediated down-regulation of proangiogenic genes constitutes a treatment strategy for the suppression of neoangiogenesis in oncology patients [17].

Arteriovenous (AV) shunts are an established model for in vivo induction of neoangiogenesis through elevated blood flow [18, 19]. Here, an AV shunt is microsurgically created by interposing a vein graft between the saphenous artery and vein on the hind limbs of a rat [20], leading to an increase in blood flow by 4.5-fold within the vascular construct [21]. Elevated shear stress on the vascular wall due to increased blood flow triggers rapid sprouting of new blood vessels from the AV shunt leading to the development of a microvascular network within 15 days [21]. Therefore, AV shunts are an ideal tool for in vivo analyses of the effects of elevated vascular shear stress on miRNA and gene expression profiles over time, which regulate flow-stimulated angiogenesis and remodeling.

**Fig. 1:**
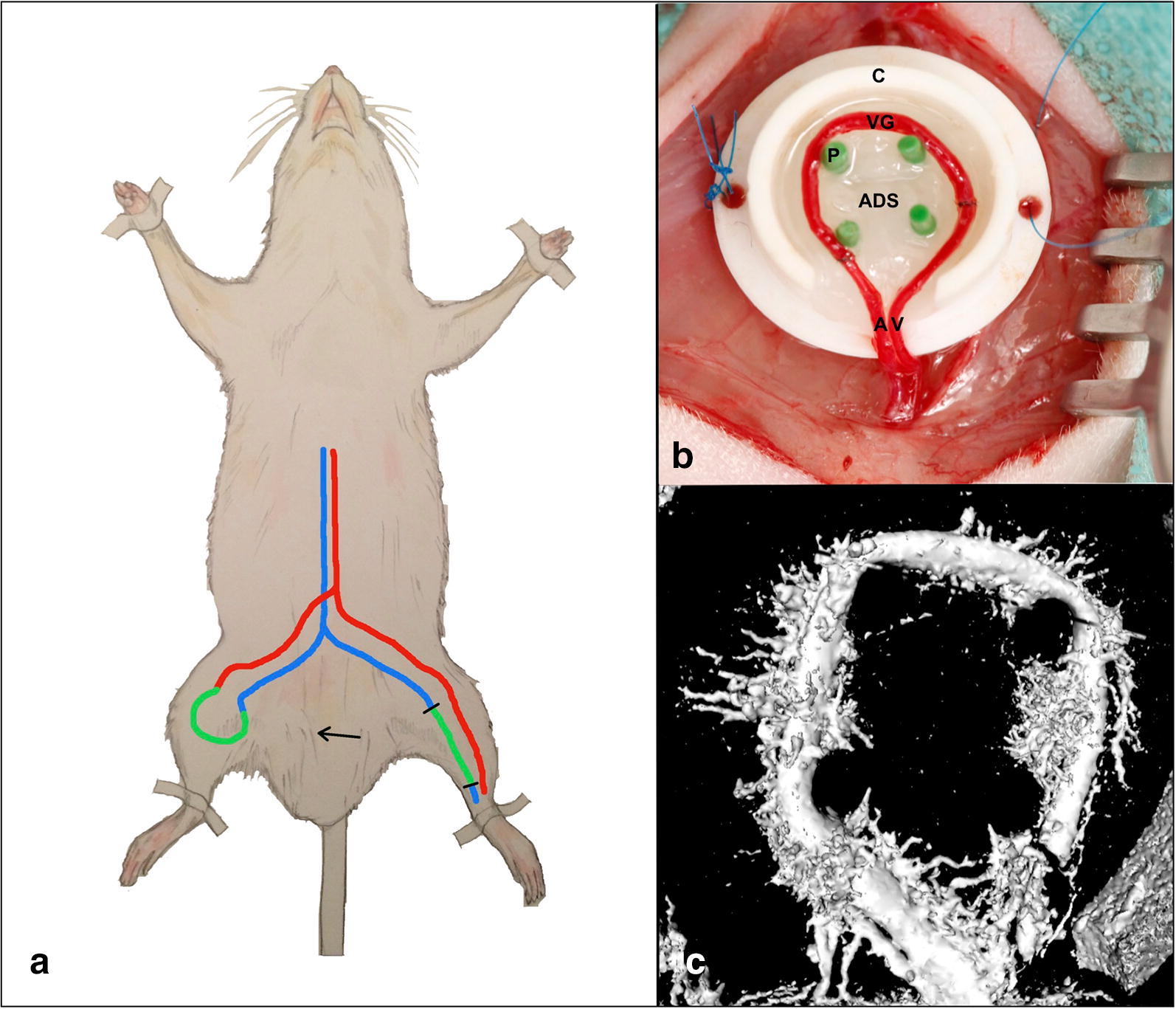
**a** Experimental setup and micro CT analysis. An arteriovenous shunt is microsurgically created on a rat’s hind limb by anastomosing a saphenous vein graft (green) from the contralateral leg between saphenous artery (red) and vein (blue). **b** The AV shunt is placed around four pins (P) for stabilization within a Teflon chamber (C). Two layers of acellular dermal substitute (ADS) are placed below and above the vascular construct (upper layer not shown). *A* saphenous artery, *V* saphenous vein, *VG* vein graft. **c** Analysis of an explanted AV shunt on postoperative day 15 by micro-computed tomography revealed a dense microvascular sprouting from the shunt vessels

We determined the expression profiles of 758 miRNAs and 30,584 messenger RNAs (mRNAs) by microarray analysis in venous tissue samples from rat AV shunts after exposure to elevated blood flow for 5, 10 and 15 days (n = 7 per group) (Fig. 1). Expression profiles were compared to sham-operated veins (end-to-end anastomosis, n = 8) in order to eliminate effects of the surgical procedure and mechanical influence caused by the operation itself on miRNA and gene expression. To identify miRNA–mRNA interactions that are relevant for the regulation of angiogenic processes, a gene ontology (GO) enrichment analysis was performed with GeneTrail2 [22, 23]. We determined miRNA/mRNA pairs with inverse correlations (r < 0.5) and a P-value < 5 × 10^−5^, which were associated with GO terms for positive, respectively negative regulation of angiogenic processes (A list of employed GO terms is shown in Additional file 1: Table S1). We used TargetScan release 7.1 for miRNA/mRNA target prediction analysis since it has proven to be the most robust prediction tool for identification of miRNA/mRNA target interactions [24, 25]. In mammals, cumulative weighted context++ scores of the binding sites are calculated and used for prediction of efficacy of targeting according to Agarwal et al. [24]. The context++ model has shown to be more predictive than any previously published model, being as predictive as most in vivo crosslinking approaches [24]. Neoangiogenesis originating from AV shunts was assessed by morphological analysis of histologic cross-sections and micro-computed tomography (micro-CT). The expression changes of strongly deregulated mRNAs were confirmed at the protein level by immunohistochemical analysis.

## Methods

### Microsurgical AV shunt creation

The experiments were performed in accordance with the German Animal Welfare Act and approved by the local governmental authorities [Landesuntersuchungsamt Rheinland-Pfalz (G15-7-047)] on 47 female Sprague–Dawley rats (Charles River Laboratories, Sulzfeld, Germany) weighing 280–320 g at ages 11–15 months. The animals had access to food and water and were kept at a 12 h dark/light cycle throughout. All surgical procedures were carried out under inhalation anesthesia with isoflurane (5% for induction and 2.5% for maintenance) in pure oxygen with a flow of 0.3 l/min. At the end of the experiments, the animals were euthanized by intracardial injection of pentobarbital under deep anesthesia. All surgical procedures were performed using a surgical microscope (magnification 16×, OPMI pico, Carl Zeiss, Germany). All rats received heparin (80 IU/kg i.v.) after completion of the anastomoses. Buprenorphin (0.05 mg/kg s.c.) was administered for analgesia preoperatively and twice daily for 2 days postoperatively. For AV shunt creation in the rats, the saphenous veins and arteries were exposed and dissected after midventral cutaneous incision on both hind limbs. A 20 mm long saphenous vein graft was harvested from the left leg and anastomosed between the right saphenous artery and vein in an end-to-end fashion, thereby creating an AV shunt (Fig. 1). The patency of both microanastomoses was assessed by observation of pulsatility and double occlusion test. Subsequently, the construct was placed into a Teflon isolation chamber (height 6 mm × diameter 12 mm, Harhaus Devices, Remscheid, Germany) between two layers of acellular dermal substitute (ADS) (MatriDerm, MedSkin Solutions Dr. Suwelack, Billerbeck, Germany) with a thickness of 2 mm. The chamber was closed with a lid (height 2 mm × diameter 14 mm) and sutured onto the underlying adductor fascia. The wounds on both thighs were subsequently closed with running subcutaneous and cutaneous sutures. The surgical technique is described elsewhere in detail [20]. For the sham operation the saphenous vein was severed and subsequently sutured by end-to-end anastomosis. Sham-operated veins were explanted on postoperative day (POD) 5 (n = 10).

### Sample collection and RNA isolation

For explantation, the Teflon chambers containing the vascular constructs were exposed by midventral incision on the rats’ hind limbs. After removal of the lid, the vessels were dissected from the surrounding ADS under the microscope, and the AV shunt was divided at the level of the two anastomoses separating the vein interposition graft from the saphenous artery and vein. The vein grafts were harvested, and care was taken to free the vessels from all attached ADS contents and suture material under the microscope. Tissue specimens were immediately placed into RNAlater solution (Applied Biosystems, Foster City, CA, USA) and stored at − 80 °C according to the manufacturer’s recommendations until further analysis. RNA isolation was performed with the Qiagen RNeasy mini Kit using the manufacturer’s protocol. Tissue samples were homogenized in 700 µl QIAzol Lysis Reagent (Qiagen, Hilden, Germany) using a TissueLyser II (Qiagen). RNA quality and quantity were assessed with a NanoDrop 2000 Spectrophotometer (Thermo Scientific, Waltham, MA, USA). All samples had a 260/280 ratio of 1.8–2.1 and were used for further analysis. RNA integrity was determined using the Agilent 6000 Nano Kit on an Agilent 2100 Bioanalyzer (Agilent Technologies, Santa Clara, CA, USA).

### MicroRNA microarray analysis

MiRNA expression profiling was performed using SurePrint™ 8 × 15 K Rat v21 miRNA microarrays (Agilent Technologies, Santa Clara, CA, USA). These microarrays contain 719 mature miRNAs of miRBase v21. All procedures were carried out according to the manufacturer’s recommendations. In brief, a total of 100 ng total RNA from each sample was dephosphorylated by incubation with calf intestinal phosphatase (CIP) at 37 °C for 30 min and denatured with 100% dimethyl sulfoxide (DMSO) at 100 °C for 7 min. Samples were labeled with pCp-Cy3 with the use of T4 ligase at 16 °C incubation for 2 h. Each labeled RNA sample was then hybridized onto an individual subarray, with each array containing probes for 306 miRNAs. Hybridizations were performed in SureHyb chambers (Agilent Technologies) at 55 °C for 20 h with rotation. Arrays were then washed, dried and scanned at a resolution of 3 μm double-pass using an Agilent G2565C Microarray Scanner. Data were acquired using Agilent AGW Feature Extraction software version 10.10.11.

### mRNA microarray analysis

MRNA expression profiling was performed using SurePrint™ 8 × 60 K G3 Rat Gene Expression v2 microarrays and one-color labeling kit (Agilent Technologies). These microarrays contained 30,584 biological features. All procedures were carried out according to the manufacturer’s protocol. In brief, 100 ng total RNA from each sample were reversely transcribed using Oligo-dT-T7 promotor primers 40 °C for 2 h to obtain cDNA. Labeled cDNA was generated using Cy3-pCp and T7 RNA polymerase at 40 °C for 2 h and subsequently purified using the RNeasy Mini kit (Qiagen, Hilden, Germany). Purified cDNA was measured with the NanoDrop 2000 Spectrophotometer (Thermo Scientific, Waltham, MA, USA) to ensure that labeled cDNA was of sufficient quality for hybridization. 600 nanograms (ng) of labeled cDNA were then hybridized onto the microarray slide at 65 °C for 17 h with 10 rpm rotation in the SureHyb chambers (Agilent Technologies). After washing and drying, the array was scanned in the Agilent G2565BA Microarray Scanner with 5 µm resolution. Data were acquired using the Agilent AGW Feature Extraction software version 10.10.11.

### Reverse transcription and quantitative real-time PCR

Quantitative real-time polymerase chain reaction (RT-qPCR) was performed to validate the microarray results on a StepOnePlus™ real-time PCR system (Applied Biosystems, Foster City, CA, USA) with the miScript PCR System along with miScript and QuantiTect Primer Assays (Qiagen, Hilden, Germany). From 29 samples used for microarray analysis, 28 samples yielded a sufficient RNA quantity allowing for RT-qPCR validation of both miRNA and mRNA expression. All steps were carried out according to the manufacturer’s recommendations. In brief, 400 ng of RNA were converted into cDNA using the miScript II RT Kit (Qiagen). During the reverse transcription step, 5× miScript HiFlex Buffer was used to promote conversion of all RNA into cDNA. The resulting cDNA was then diluted to have 0.5 ng/µl for miRNA and 2.5 ng/µl for mRNA. All reverse transcription PCR (RT-PCR) experiments were performed using the QIAgility™ automated PCR setup (Qiagen) before performing RT-qPCR analysis on a StepOnePlus™ Real-Time PCR system (Applied Biosystems). Briefly, each miRNA PCR reaction contained 2 µl cDNA, 10 µl QuantiTect SYBR Green PCR Mix, 2 µl miScript Universal Primer and 2 µl miScript Primer Assays for rno-miR-340-5p, rno-miR-19b-3p, rno-miR-223-3p, rno-miR-31a-5p, rno-miR-210-3p, rno-let-7b-5p, and rno-let-7c-5p plus RNase-free water to a total volume of 20 µl, and was placed into an individual well of a 96-well plate. Each mRNA PCR reaction contained, 2 µl cDNA, 10 µl QuantiTect SYBR Green PCR Mix and 2 µl QuantiTect Primer Assay for hypoxia-inducible factor 1-alpha (HIF1A), toll like receptor 6 (TLR6), vascular endothelial growth factor A (VEGFA), thrombospondin 3 (THBS3), and *N*-myc downstream regulated gene 2 (NDRG2) plus RNase-free water amounting to a total volume of 20 µl, and was placed into an individual well of a 96-well plate. Reactions were run with the following thermal cycling parameters: initial activation step 95 °C for 15 min followed by 40 cycles at 94 °C for 15 s (denaturation), 55 °C for 30 s (annealing), and 70 °C for 30 s (extension). Then final dissociation curves (melting curves) were made and PCR plates were kept at 4 °C until they were taken out of the PCR machine. QuantiTect Primer Assays for hypoxanthine-guanine phosphoribosyltransferase (HPRT) and glyceraldehyde 3-phosphate dehydrogenase (GAPDH) were chosen as reference genes for mRNA normalization, and rno-miR-93-5p and rno-RNU6B were chosen as endogenous controls for miRNA normalization. In addition, a no template control (NTC) and no reverse transcriptase control (NRT) were included in each run. All RT-qPCR experiments were performed in triplicate.

### Histological analysis

In order to visualize the expanding functional vasculature around the AV shunts over time and analyze endothelial protein expression, a histological analysis of AV shunts and control veins was performed in 17 animals (POD 5: n = 3, POD 10: n = 6, POD 15: n = 6, control veins: n = 2). Functional vessels on histologic cross-sections were highlighted by perfusion with black India ink (Windsor & Newton, London, England). After abdominal incision the distal descending aortas of the rats were cannulated with a 24-gauge catheter and flushed with heparin solution (100 IU/ml) followed by 30 ml of warm Indian ink solution. Subsequently the constructs were explanted and fixed in 4% paraformaldehyde (PFA) in phosphate-buffered saline (PBS), followed by dehydration and embedding into paraffin. Histological cross sections were obtained perpendicularly to the longitudinal main vessel axis. Hematoxylin and eosin staining was performed according to standard protocols. Stained slides were visualized by bright field microscopy and recorded using with the Axio Vision 4 software (Carl Zeiss Microscopy, Jena, Germany).

### Immunohistochemistry

Serial sections of 4 µm thickness were generated and immunostained for C–X–C motif chemokine ligand 2 (CXCL2) (unconjugated rabbit polyclonal anti-CXCL2 antibody, LS-C-415005-100, LifeSpan BioSciences, Seattle, WA, USA) and IL1A (anti-IL1A primary antibody, OAAN00770, Aviva Systems Biology, San Diego, CA, USA). Staining was performed using a BenchMark XT immunostainer (Roche Diagnostics, Germany). An UltraView DAB detection and amplification kit (Roche Diagnostics, Germany) was used and the slides were counterstained with hematoxylin for 4 min and post-counterstained with bluing agent for 4 min. Then, the slides were washed and dehydrated in 70% to 100% reagent alcohol baths and xylenes baths before applying coverslips. Standardized images of one complete vessel cross section were obtained at 63× magnification (Axio Vision 4, Carl Zeiss Microscopy) under standardized conditions for white balance and exposure time (161.2 ms). The images were cropped to non-overlapping areas of 165 × 220 µm covering the complete endothelium. Quantitative assessment of endothelial protein expression was performed with the freeware ImageJ (NIH, Bethesda, MD, USA, https://imagej.nih.gov). Images were converted into grayscale and per cross section 24 ECs were manually selected as regions of interest (ROI). Integrated Density (defined as the product of area (µm^2^) and mean gray value) was assessed in each of the 24 ROIs and means were calculated. Average integrated densities of all animals per group were then compared between the groups.

### Micro-computed tomography

For micro-computed tomography (micro-CT), 20 ml Microfil Silicone Rubber (MV-122, Flow Tech, Carver, MA, USA) containing 5% of MV Curing Agent (Flow Tech) was applied instead of India ink in one animal (POD 15). A customized Micro-CT scanner (Y.Fox, Yxlon, Garbsen, Germany) with an open multifocus X-ray tube (10–160 kV; focal spot sizes 1–5 μm), a CNC manipulator, and a 14-bit direct amorphous silicon flat panel detector (Varian PaxScan 2520 D/CL; Varian, Palo Alto, CA, USA) was used. Data were analyzed with Osirix v. 4.1.1 (Pixmeo, Geneva, Switzerland).

### Bioinformatics analysis

Statistical analysis was performed using R (versions 3.3.2/3.3.3; http://www.r-project.org). Samples from sham-operated rats served as controls. Raw data generated by the Agilent Feature Extraction image analysis software was quantile normalized, and signal intensities of both miRNAs and mRNAs were log_2_-transformed before further analysis. P-values were corrected for multiple testing using the Benjamini–Hochberg procedure. For RT-qPCR, the DataAssist™ Software v3.0 (Applied Biosystems) was used to calculate the fold-changes in miRNA and mRNA expression by the equation 2^−ΔΔCT^ [26]. Correlation of miRNA and mRNA data was performed with the Spearman correlation. Student’s T-test was used to compare mean Integrated Densities of immunohistochemical analysis.

## Results

### MicroRNA microarray analysis

**Fig. 2:**
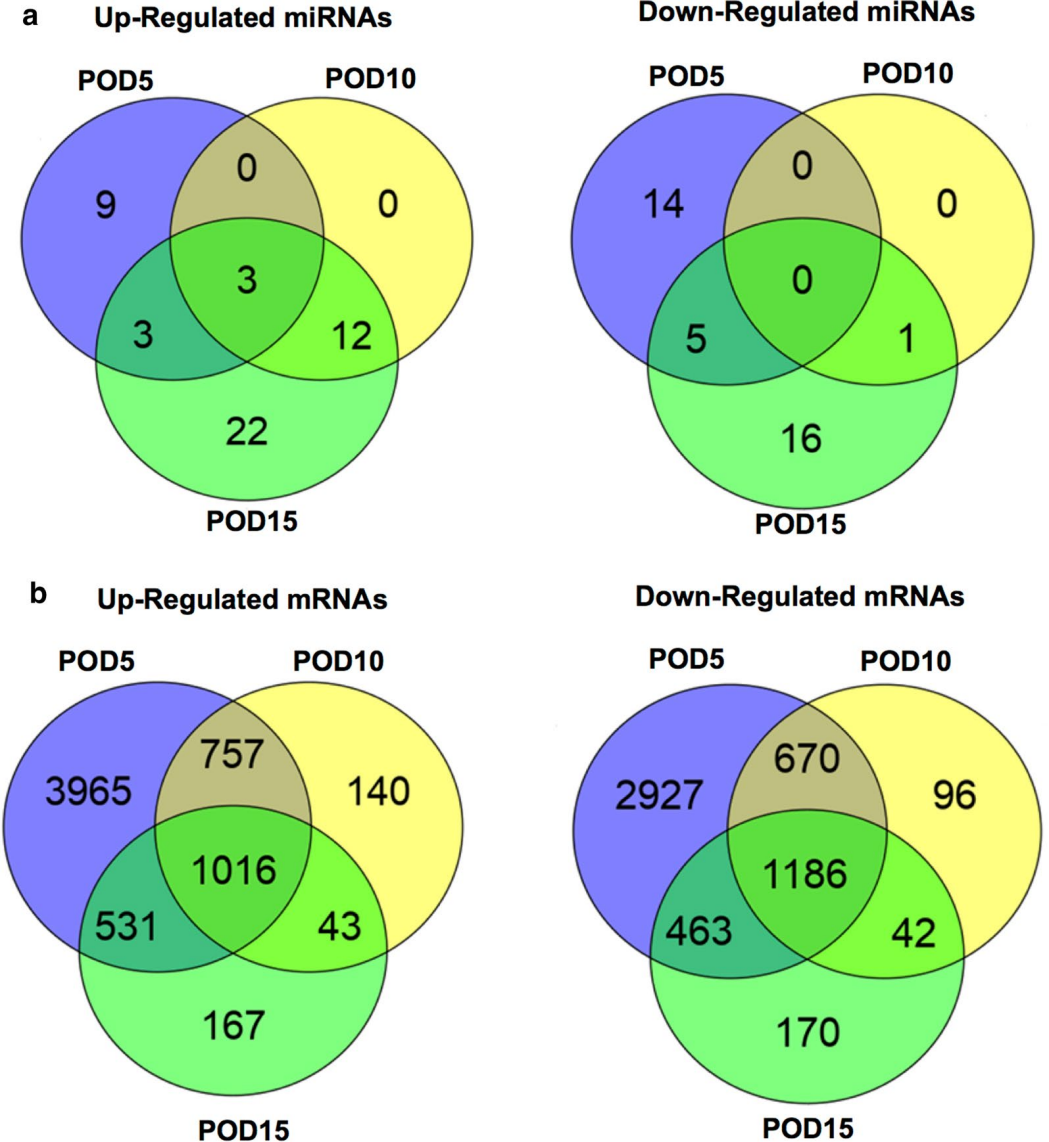
Deregulated microRNAs and messenger RNAs. Venn diagrams showing numbers of up- and down-regulated microRNAs (miRNAs) (**a**), as well as of up- and down-regulated messenger RNAs (mRNAs) (**b**) in the examined groups compared to controls. *POD* postoperative day

Microarray analysis of samples from AV shunts explanted on POD 5 revealed that 15 miRNAs were up-regulated and 19 miRNAs were down-regulated as compared to controls. On POD 10, 15 miRNAs were up-regulated, whereas only miR-203a-3p was down-regulated. The strongest deregulation of miRNA expression was observed on POD 15 with 40 miRNAs being up-regulated and 16 miRNAs being down-regulated, respectively. A bimodal distribution of miRNA expression was observed across the different groups: Specific miRNAs were deregulated only in the early postoperative period (POD 5) with 9 miRNAs being up- and 14 miRNAs being down-regulated. A different subset of miRNAs was deregulated in the late postoperative period, with 22 miRNAs being up-regulated and 16 miRNAs being down-regulated only on POD 15. Three miRNAs, namely miR-19b-3b, miR-223-3p, and miR-340-5p, were up-regulated in all groups (fold change (FC) > 1.5, P < 0.05 for all comparisons) (Fig. 2a). A complete list of significantly deregulated miRNAs is shown in Additional file 1: Table S2.

### mRNA microarray analysis

Microarray analysis of mRNA expression revealed the strongest deregulation on POD 5, with 6269 mRNAs being up-regulated and 5246 mRNAs being down-regulated respectively. On POD 10, 1956 mRNAs were up-regulated and 1994 mRNAs were down-regulated. In contrast to miRNA expression, which exhibited a stronger deregulation on POD 15 compared to POD 10, comparable amounts of deregulated mRNAs were observed on POD 15 with 1757 mRNAs being up-regulated and 1861 mRNAs being down-regulated, respectively. In all groups, 1016 mRNAs were up-regulated, whereas 1186 mRNAs were down-regulated (Fig. 2b). Among the deregulated mRNAs in samples from AV shunts, a marked over-expression of cytokines, especially chemokines, interleukins, and tumor necrosis factor (TNF) associated genes was observed. Moreover, a striking up-regulation of oxygenation-associated genes [HIF1A, heme oxygenase 1 (HMOX1)] as well as angiopoetic growth factors [VEGFA, platelet derived growth factors (PDGF)] and their downstream signaling factors was observed across all groups. We observed a significant down-regulation of antiangiogenic proteins in AV shunts: angiotensinogen (AGT) and angiotensinogen converting enzyme (ACE), as well as the thrombospondins 3 and 4 (THBS3, THBS4) were continuously down-regulated. Forkhead box C1 (FOXC1), synaptojanin-2 binding protein (SYNJ2BP) and delta-like 1 (DLL1) were down-regulated in all groups as well. Endothelial nitric oxide synthase (NOS3, eNOS) was significantly downregulated on POD 15. On POD 5 and 10 negative fold-changes were observed for eNOS but statistical significance was not met. For Kruppel-like factor 2 (KLF2), a significant downregulation was found on POD 5 (FC: − 2.25), whereas statistical significance was not met on POD 10 and 15, however fold-changes were also negative. Detailed FCs of selected mRNAs are shown in Additional file 1: Table S3 (P < 0.05 for all comparisons).

### Correlation of miRNA and mRNA microarray data

**Fig. 3:**
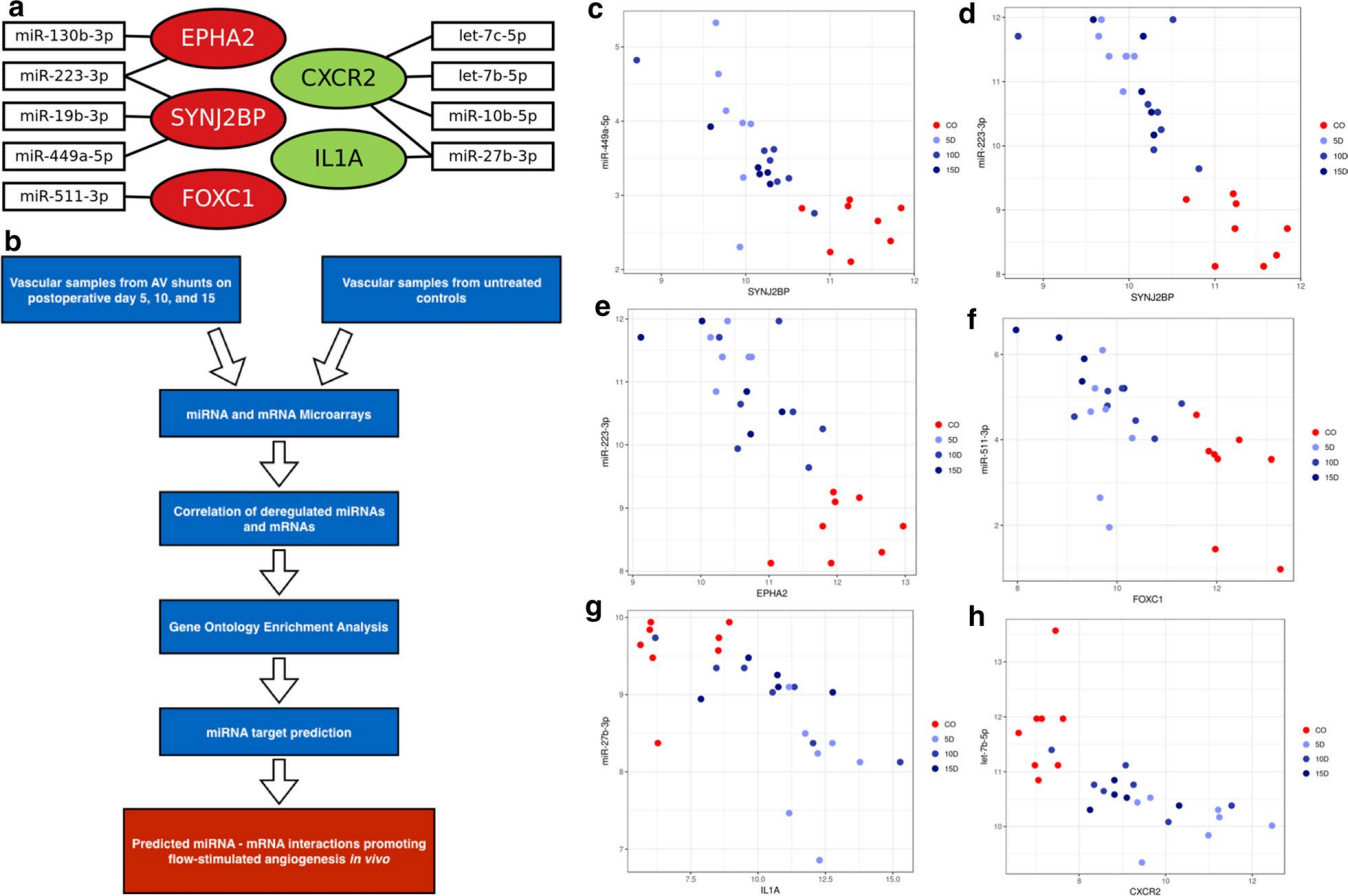
**a** Inverse correlations of microRNAs and messenger RNAs with relevance to angiogenesis. Deregulated microRNAs (miRNAs) and messenger RNAs (mRNAs) with significant inverse correlations as well as association with angiogenesis-related gene ontology (GO) terms and predicted target interactions according to TargetScan. Green background: up-regulated mRNAs. Red background: down-regulated mRNAs. Connections between miRNAs and mRNAs represent significant inverse correlations (r < − 0.5, P < 5 × 10^−5^). **b** Schematic workflow of the study for identification of miRNA-regulated pathways in flow-stimulated angiogenesis. Spearman correlation plots show significant inverse correlations between the expression levels of synaptojanin-2 binding protein (SYNJ2BP) and miR-449-5p (**c**) as well as miR 223-3p (**d**), ephrin receptor kinase 2 (EPHA2) and miR-223-3p (**e**), forkhead box C1 (FOXC1) and miR-511-3p (**f**), interleukin-1 alpha (IL1A) and miR-27b-3p (**g**), as well as C–X–C chemokine receptor 2 and let-7b-5p (**h**)

**Fig. 4:**
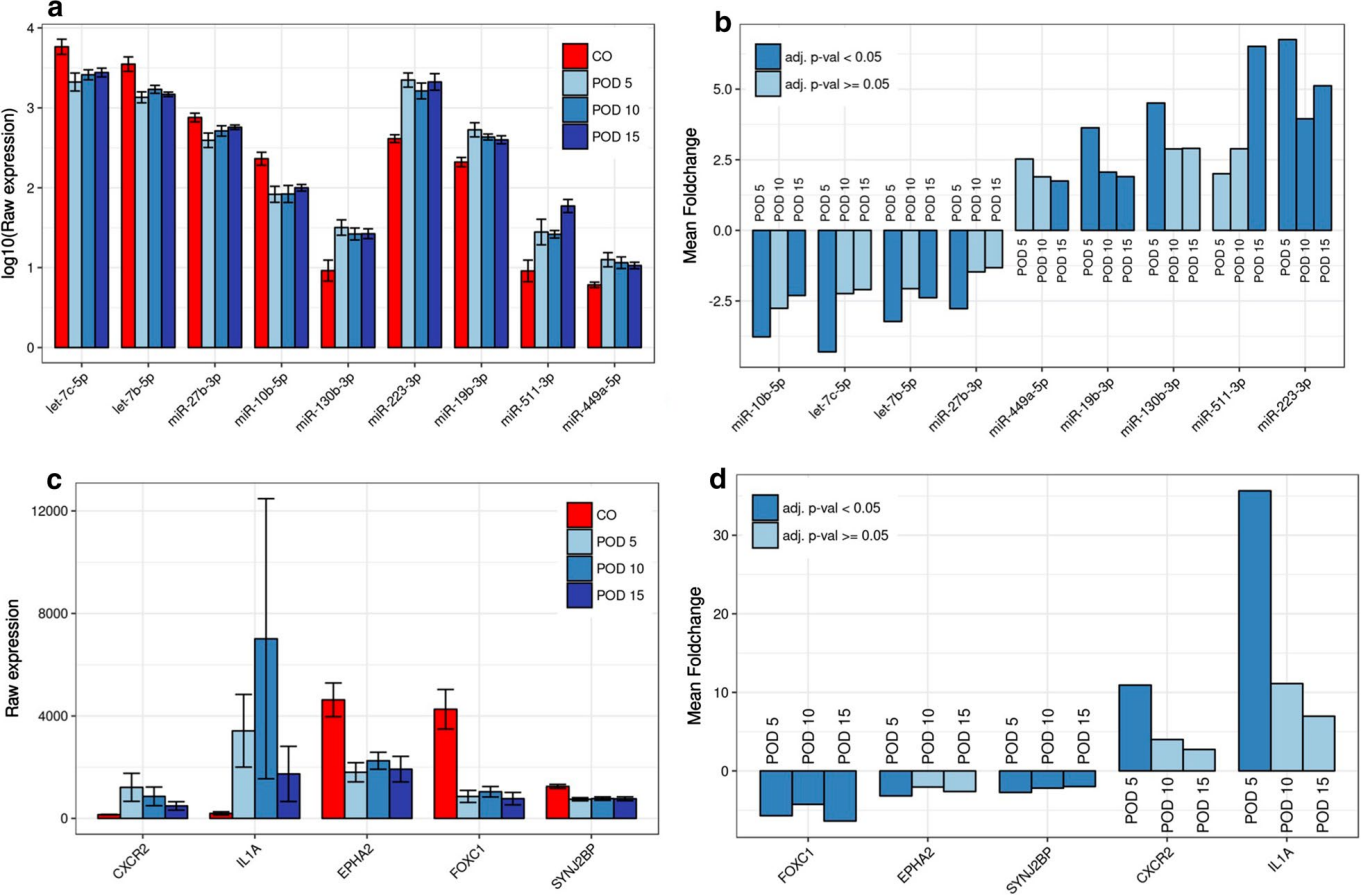
Differential expression of microRNAs and messenger RNAs with relevance to angiogenesis. Mean expression levels ± 1 SEM (**a**) and mean fold-changes (**b**) compared to controls (CO) for microRNAs (miRNAs) with significant inverse correlations and target interactions with messenger RNAs (mRNAs) (**c**, **d**) in the analyzed groups. Mean expression levels for miRNAs are shown log10-transformed for illustrative purposes

In order to identify miRNA-regulated pathways of flow-stimulated angiogenesis, a correlation analysis of miRNA and mRNA expression data was performed. On POD 5, 7495 miRNA/mRNA pairs with a negative correlation were found (P < 0.05; r ≤ − 0.5). On POD 10 and 15, 969 respectively 2253 deregulated miRNA/mRNA pairs with negative correlations were evident (P < 0.05; r ≤ − 0.5). In the POD 5 group, 48 negatively correlating miRNA/mRNA pairs were identified, for which the respective mRNAs were up-regulated and associated with GO terms for positive regulation of angiogenic processes (i.e., up-regulated inducers of angiogenesis). In five of these inversely correlating miRNA/mRNA pairs, the mRNA was identified as a predicted target of the respective miRNA according to TargetScan: C–X–C chemokine receptor 2 (CXCR2) with let-7c-5p, let-7b-5p, miR-27b-3p, and miR10b-5p, respectively, as well as interleukin-1 alpha (IL1A) with miR-27b-3p. Nine negatively correlating miRNA/mRNA pairs were found on POD 5, for which the respective mRNAs were down-regulated and associated with GO terms for negative regulation of angiogenesis (i.e. down-regulated inhibitors of angiogenesis). In four of these pairs, specific target interactions were present: SYNJ2BP and miR-223-3p, and miR-19b-3p as well as ephrin receptor 2 alpha (EPHA2) and miRNAs 130b-3p, and 223-3p respectively. On POD 10, six negatively correlating miRNA/mRNA pairs were found, for which mRNAs were down-regulated and associated with GO terms for negative regulation of angiogenesis. Predicted target interactions were present between SYNJ2BP and miR-223-3p as well as miR-19b-3p. In the POD 15 group, seven negatively correlating miRNA/mRNA pairs were identified, for which the respective mRNAs were up-regulated and associated with GO terms for positive regulation of angiogenesis. However, none of these pairs were predicted targets according to TargetScan. We identified 14 negatively correlating miRNA/mRNA pairs on POD 15, for which the respective mRNAs were down-regulated and associated with GO terms for negative regulation of angiogenesis. Among these pairs, the predicted target interactions of SYNJ2BP and miRNAs 223-3p and 19b-3p was also present. Moreover, predicted interactions between SYNJ2BP and miR-449a-5p as well as between FOXC1 and miR-511-3p were observed (r < − 0.5, P < 5 × 10^−5^ for all correlations) (Table 1, Figs. 3, 4).

**Table 1:** Inverse correlations between micro-RNAs and up-regulated messenger RNAs (mRNA) which induce angiogenesis as well as down-regulated mRNAs which inhibit angiogenesis

miRNA	Gene symbol	Mean fold-change miRNA	Mean fold-change mRNA	GO term	Correlation	P-value	Weighted context++ score	Weighted context++ score percentile
mRNAs inducing angiogenesis								
POD 5								
let-7c-5p	CXCR2	− 4.30	10.92	0045766	− 0.66	3.31 × 10^−6^	− 0.193	79
let-7b-5p	CXCR2	− 3.23	10.92	0045766	− 0.64	3.47 × 10^−6^	− 0.157	74
miR-27b-3p	CXCR2	− 2.77	10.92	0045766	− 0.6	1.50 × 10^−5^	− 0.157	91
miR-10b-5p	CXCR2	− 3.76	10.92	0045766	− 0.57	3.58 × 10^−5^	− 0.414	98
miR-27b-3p	IL1A	− 2.77	35.67	0045766	− 0.59	1.82 × 10^−5^	− 0.203	94
mRNAs inhibiting angiogenesis								
POD 5								
miR-130b-3p	EPHA2	4.51	− 3.18	0016525	− 0.61	9.76 × 10^−6^	− 0.069	73
miR-223-3p	EPHA2	6.76	− 3.18	0016525	− 0.6	1.46 × 10^−5^	− 0.134	86
miR-223-3p	SYNJ2BP	6.76	− 2.74	1903671	− 0.73	1.85 × 10^−7^	− 0.013	37
miR-19b-3p	SYNJ2BP	3.63	− 2.74	0016525	− 0.58	2.96 × 10^−5^	− 0.052	52
POD 10								
miR-19b-3p	SYNJ2BP	2.06	− 2.19	0016525	− 0.58	2.96 × 10^−5^	− 0.052	52
miR-223-3p	SYNJ2BP	3.95	− 2.19	0016525	− 0.73	1.85 × 10^−7^	− 0.086	77
POD 15								
miR-511-3p	FOXC1	6.52	− 6.39	0016525	− 0.56	4.33 × 10^−5^	− 0.038	62
miR-223-3p	SYNJ2BP	5.12	− 1.99	0016525	− 0.73	1.85 × 10^−7^	− 0.086	77
miR-449a-5p	SYNJ2BP	1.75	− 1.99	0016525	− 0.64	5.74 × 10^−7^	− 0.143	74

Weighted context++ scores and percentiles were calculated with TargetScan (release 7.1)

*GO* gene ontology, *POD* postoperative day, *CXCR2* C–X–C motive chemokine receptor 2, *IL1A* interleukin 1 alpha, *SYNJ2BP* synaptojanin-2 binding protein, *EPHA2* ephrin receptor 2 A, *FOXC1* forkhead box C1

### Validation of selected miRNAs and mRNAs by RT-qPCR

In order to validate the microarray data by RT-qPCR we selected three miRNAs (miR-19b-3p, miR-340-5p, and miR-223-3p), that were significantly up-regulated in all examined groups (POD 5, 10, and 15) as well as miR-31a-5p which was up-regulated on POD 10 and POD 15 and miRNA miR-210-3p which was up-regulated only on POD 15. Moreover, we chose let-7c-5p, which was significantly down-regulated only on POD 5, and let-7b-5p, which was down-regulated on POD 5 and 15. We selected two key regulators of angiogenesis, namely VEGFA and HIF1A [27], which were up-regulated on POD 5 and 10, as well as TLR6, which was up-regulated on POD 5 and 15. Moreover, we selected NDRG2 and THBS3, which were down-regulated in all groups.

Aliquots from the same samples used for microarray analysis were used for RT-qPCR validation (POD 5: n = 3, POD 10: n = 6 and POD 15: n = 7; controls: n = 7). Due to the small size of the tissue specimens, four samples from the POD 5 group and one sample from the POD 10 group yielded not enough RNA for RT-qPCR analysis. For each of the seven miRNAs, except for miR-210-3p and miR-31a-5p in the POD 5 group, RT-qPCR analysis revealed concordant expression changes to the microarray analysis in all groups, e.g. miR-223-3p, miR-19b-3p and miR-340-5p showed an increased expression in all groups (POD 5, 10, 15) compared to controls in both microarray and RT-qPCR analysis. Likewise, let-7b-5p and let-7c-5p showed a reduced expression across all groups (Table 2).

**Table 2:** Quantitative real-time polymerase chain reaction (qPCR) and miRNA microarray analysis for selected miRNAs

miRNA	qPCR			miRNA microarray		
	Fold change	P-value	Regulation	Fold change	P-value	Regulation
POD 5						
miR-19b-3p	1.40	0.2180	Up	3.63	0.0243	Up
miR-210-3p	− 1.3	0.2350	Down	3.90	0.1504	Up
miR-223-3p	2.78	0.2260	Up	6.75	6.67 × 10^−6^	Up
miR-31a-5p	− 1.08	0.8740	Down	3.37	0.4048	Up
miR-340-5p	1.61	0.2760	Up	3.32	0.0087	Up
let-7b-5p	− 2.04	0.2200	Down	− 3.23	0.0144	Down
let-7c-5p	− 7.14	0.0040	Down	− 4.30	0.0130	Down
POD 10						
miR-19b-3p	1.84	0.0600	Up	2.06	0.0364	Up
miR-210-3p	1.75	0.0110	Up	5.24	0.1289	Up
miR-223-3p	3.25	0.0630	Up	3.95	0.0266	Up
miR-31a-5p	6.87	0.0030	Up	18.49	0.0097	Up
miR-340-5p	3.23	0.0490	Up	2.82	0.0193	Up
let-7b-5p	− 2.22	0.0080	Down	− 2.07	0.1736	Down
let-7c-5p	− 2.38	0.0110	Down	− 2.24	0.1611	Down
POD 15						
miR-19b-3p	2.36	0.1390	Up	1.90	0.0464	Up
miR-210-3p	1.97	0.0050	Up	5.85	0.0450	Up
miR-223-3p	3.17	0.0050	Up	5.12	0.0236	Up
miR-31a-5p	9.74	0.0080	Up	22.08	0.0046	Up
miR-340-5p	3.80	0.0570	Up	3.74	0.0089	Up
let-7b-5p	− 1.75	0.0420	Down	− 2.39	0.0458	Down
let-7c-5p	− 1.75	0.0540	Down	− 2.10	0.1069	Down

*POD* postoperative day

For mRNAs, RT-qPCR analysis uniformly revealed concordant expression changes to the microarray analysis across all examined groups. In accordance with microarray data VEGFA, HIF1A and TLR6 were up-regulated, whereas NDRG2 and THBS3 were down-regulated in all groups.

The miRNA and mRNA expression changes in AV shunts compared to controls were concordant throughout for all miRNAs and mRNAs, yet were partly below the significance threshold, apparently due to the reduced sample size in the RT-qPCR analysis, especially in the POD 5 group. Having encountered no contradictory results as to miRNA and mRNA expression changes, we did not opt to increase the sample size, i.e., the number of sacrificed rats, for RT-qPCR validation purposes alone, in accordance with the Animal Welfare Act. In summary, RT-qPCR analysis corroborated the results of the microarray analysis for both miRNAs and mRNAs in terms of concordant expression changes across all examined groups (Table 3).

**Table 3:** Quantitative real-time polymerase chain reaction (qPCR) and mRNA microarray analysis for selected mRNAs

mRNA	qPCR			miRNA microarray		
	Fold change	P-value	Regulation	Fold change	P-value	Regulation
POD 5						
HIF1A	2.18	0.214	Up	1.97	0.0003	Up
TLR6	4.00	0.071	Up	4.11	0.0003	Up
VEGFA	3.45	0.22	Up	4.41	0.0007	Up
THBS3	− 14.29	0.001	Down	− 8.78	5.33 × 10^−6^	Down
NDRG2	− 33.33	0.002	Down	− 21.00	0.0005	Down
POD 10						
HIF1A	1.69	0.049	Up	1.59	0.0380	Up
TLR6	2.72	0.061	Up	2.22	0.1089	Up
VEGFA	3.46	0.015	Up	3.12	0.0379	Up
THBS3	− 7.14	0.001	Down	− 1.36	0.0050	Down
NDRG2	− 7.69	0.003	Down	− 10.91	0.0157	Down
POD 15						
HIF1A	1.19	0.288	Up	1.38	0.3585	Up
TLR6	2.22	0.003	Up	2.46	0.0106	Up
VEGFA	2.32	0.066	Up	2.71	0.1847	Up
THBS3	− 11.11	0.001	Down	− 1.57	0.0008	Down
NDRG2	− 11.11	0.003	Down	− 8.48	0.0002	Down

*POD* postoperative day, *HIF1A* hypoxia-inducible factor-1 A, *TLR* 6 toll-like receptor 6, *VEGFA* vascular endothelial growth factor-A, *THBS3* thrombospondin 3, *NDRG2* NMYC downstream-regulated gene 2, *POD* postoperative day

### Histologic analysis and micro-CT

**Fig. 5:**
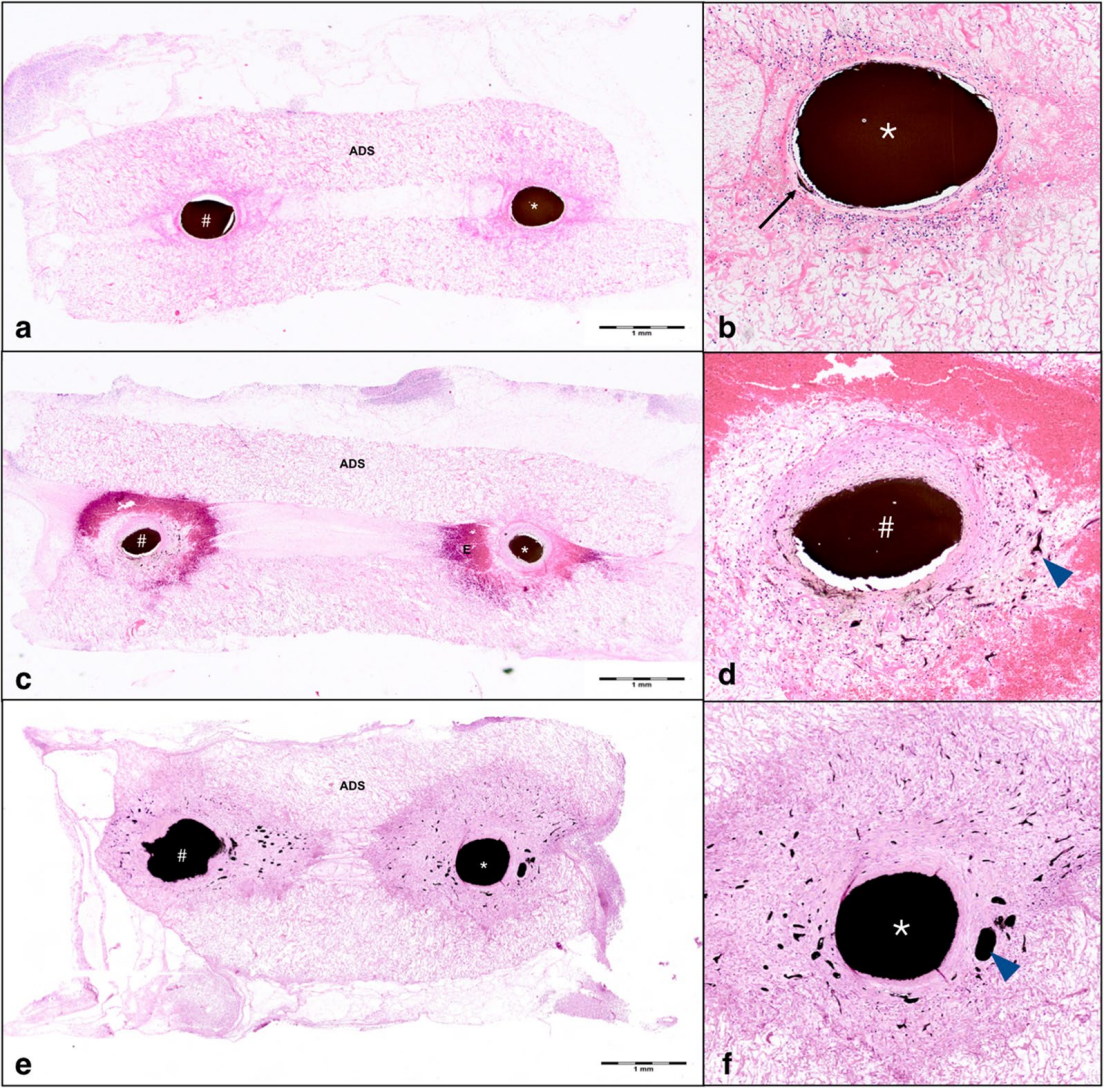
Histologic analysis of arteriovenous shunts. Histologic analysis of hematoxylin/eosin stained cross sections of explanted vascular constructs on postoperative day (POD) 5 (**a**, **b**), 10 (**b**, **c**), and 15 (**d**, **e**) show ink-perfused vessel lumina of both saphenous artery (*) and vein (#). Early intramural vessel sprouting was detected within the wall of the saphenous artery (*) on POD 5 (**b**, black arrow). Neoangiogenesis within the acellular dermal matrix (ADS) surrounding the saphenous vein (#) was clearly visible on POD 10 (blue arrowheads, **d**). Neoangiogenesis had strongly expanded and surrounded both artery (*) and vein (#) on POD 15 (**e**, **f**, blue arrowheads). An accumulation of erythrocytes (E) around the main vessels was seen on POD 10 (**c** and **d**)

Histologic analysis of hematoxylin/eosin-stained cross-sections of paraffin-embedded AV shunt constructs revealed early intramural vessel sprouting on POD 5 (Fig. 5a). Neoangiogenesis within the acellular matrix surrounding both saphenous artery and vein was clearly visible on POD 10. An accumulation of erythrocytes around the areas of neoangiogenesis was visible on POD 10, likely due to a high intraluminal pressure during ink perfusion of the AV shunts, causing cell leakage through the fragile vascular walls of the developing vasculature (Fig. 5b). On POD 15, neoangiogenesis had further expanded and spanned an area equaling the diameter of the main vessels (Fig. 5c). Analysis of micro-CT images confirmed a dense microvascular sprouting from the AV shunt vessels on POD 15 (Fig. 1 c).

### Immunohistochemistry

**Fig. 6:**
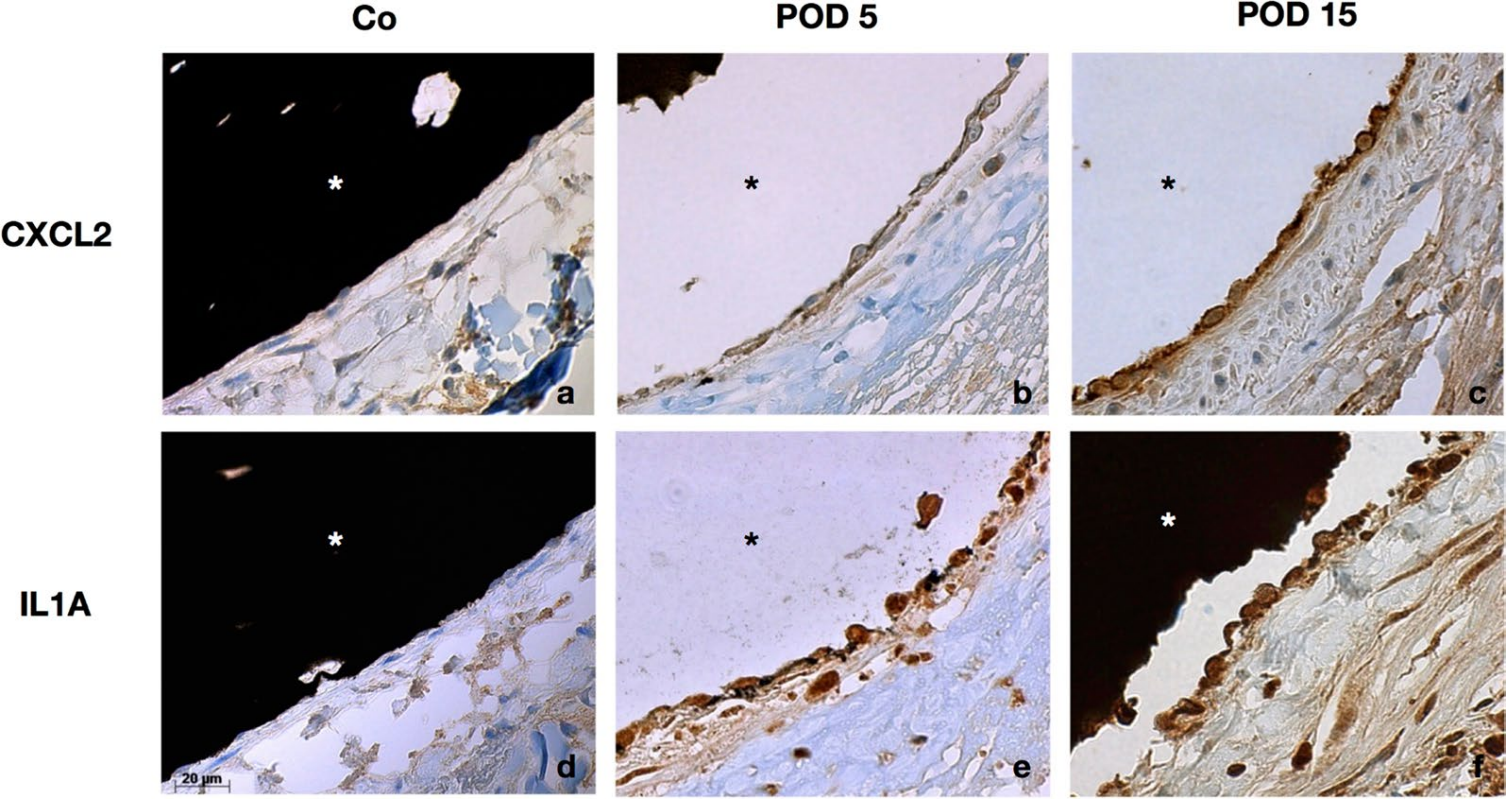
Immunohistochemical staining for CXCL2 and IL1A. Immunohistochemical analysis of C–X–C chemokine ligand 2 (CXCL2) (**a**–**c**) and interleukin-1 alpha (IL1A) protein expression (**d**–**f**) in AV shunt and control vein (Co) cross sections show a significant increase in the expression of both proteins in the endothelial cells of AV shunts on postoperative day (POD) 5 (**a**, **d**) and an even stronger expression on POD 15 (**c**, **f**) compared to control veins (**a**, **d**). The black Indian ink filling the vessel lumen (*) appears detached from the endothelium in some sections due to cutting artifacts

Based on the results of the microarray analysis showing a strong overexpression of the cytokines CXCL2 and IL1A at the mRNA level, we selected these two genes for further validation at the protein level. Immunohistochemical analysis of IL1A and CXCL2 expression in AV shunt and control vein cross sections showed a significant increase in CXCL2 and IL1A expression in the endothelial cells of AV shunts on POD 5, 10 and 15 compared to control veins. Comparison between AV shunt subgroups revealed a significant increase in expression for both proteins at POD 15 compared to POD 5 and 10 respectively. Expression of CXCL2 at POD 10 had decreased significantly compared to POD 5, whereas no significant differences between POD 5 and 10 were evident for IL1A (Table 4, Fig. 6).

**Table 4:** Immunohistochemical analysis of C–X–C chemokine ligand 2 (CXCL2) and interleukin-1 alpha (IL1A) protein expression in AV shunt and control vein (Co) cross sections

Group	Mean integrated density, µm^2^ × pixel (SEM) Endothelium		P (vs. Co) CXCL2	P (vs. POD 5) CXCL2	P (vs. POD 10) CXCL2	P (vs. POD 15) CXCL2
	CXCL2	IL1A	IL1A	IL1A	IL1A	IL1A
Co	5173 (351)	2987 (331)		0.0005	10^−5^	9 × 10^−15^
				0.02	0.003	6 × 10^−6^
POD 5	7838 (559)	5186 (501)	0.0005		0.01	0.03
			0.02		0.21	0.0003
POD 10	6473 (265)	4590 (207)	10^−5^	0.01		7 × 10^−10^
			0.003	0.21		10^−10^
POD 15	9251 (345)	7606 (400)	9 × 10^−15^	0.03	7 × 10^−10^	
			6 × 10^−6^	0.0003	10^−10^	

*POD* postoperative day, *SEM* standard error of the mean

## Discussion

Angiogenesis occurs either as a physiological response of the organism to locally challenged blood supply after either acute (trauma, embolism) or chronic (atherosclerosis) damage, or as a pathological process triggered by proangiogenic factors secreted from tumor cells [28]. Due to the limited recovery time granted by malnourished tissue, neoangiogenesis must be a rapid yet well-orchestrated process of three-dimensional proliferation and differentiation of ECs and VSMCs interacting with the surrounding tissue in need of supply. Mechanical stimuli indicating critical dysfunction of existing vessels are transferred via mechanosensory proteins and signaling cascades into expression of proangiogenic factors [29]. Studies on cell cultures have also identified complex networks of miRNAs governing rapid changes of gene activities during this process [10]. In vitro studies, even if three-dimensional cell culturing techniques are employed [30] are unable to represent the molecular picture of neoangiogenesis in its full complexity, as they can neither properly simulate the continuously changing fluid mechanics nor the multitude of interacting cell types within an emerging vascular network. This is why we present, to the best of our knowledge, the first in vivo study investigating the response of biochemical pathways to elevated vascular shear stress and their interaction with miRNAs over several phases of neoangiogenesis.

Our data show strong and significant changes in miRNA and gene expression profiles over time within the vascular wall of AV shunts compared to sham-operated veins. Since the only difference between treatment and control groups in our experiment was the presence of an elevated blood flow in the treatment group, the observed differences in miRNA expression are likely evoked by elevated vascular shear stress within AV shunts. Notably, changes in miRNA and gene expression patterns precede the sprouting of new blood vessels from AV shunts, since significant deregulations of miRNA as well as mRNA expression arise already on POD 5, when functional microvasculature is not yet present around the main vessels. This finding demonstrates that alterations in molecular signaling in response to elevated blood flow occur rapidly and are already detectable when the physical outgrowth of blood vessels is still limited to intramural sprouting of new blood vessels within the vascular wall of the main vessels (Fig. 5a).

### Cytokines

Cytokines, among them chemokines, interleukins, and the TNF family, play a well-established role in the early induction of angiogenesis through up-regulation of growth factors like VEGFA [31]. C–X–C and C–C motif chemokines showed a strong mRNA overexpression in AV shunts. CXCL2, 3, and 11, as well as CCL6 and 9 were significantly upregulated on POD 5 compared to controls. We confirmed CXCL2 overexpression in ECs of AV shunts on the protein level on POD 5, 10 and 15 compared to control veins by immunohistochemical staining. CXCL2 was upregulated on the mRNA level in all AV shunt groups, however, statistical significance was missed on POD 10 (FC: 11, adjusted P: 0.25) and POD 15 (FC: 13, adjusted P: 0.13) which may be related to the relatively low sample size. In wound healing, CXCL2 is up-regulated 3 days after skin incision and is also involved in bronchial angiogenesis [32, 33]. Studies on peripheral blood cells and murine skin grafts subjected to extracorporeal shock wave treatment demonstrated a relationship between neoangiogenesis and a mechanically induced cytokine up-regulation [34, 35]. Our in vivo data indicate that elevated vascular shear stress leads to a strong up-regulation of chemokines as well as their receptors within the vascular wall, which seem to determinants of flow-stimulated angiogenesis.

Among the down-regulated miRNAs on POD 5, let-7b-5p, let-7c-5p, miR-27b-3p, and miR-10b-5p exhibited inverse correlations with C–X–C motif chemokine receptor 2 (CXCR2), which we found to be 11-fold up-regulated and a predicted target of these miRNAs. CXCR2 plays a pivotal role in angiogenesis by mediating recruitment of endothelial progenitor cells via its ligands CXCL1 and CXCL2 and acts as a receptor to several CXCL-chemokines, among them CXCL2 and CXCL3 [36], sharing its expression profile over time with them in our study. Moreover, CXCR2 stimulates cell migration via adaptor protein 2 (AP2) [37], which we found to be up-regulated on POD 5 and 10.

Our data indicate that elevated shear stress in AV shunts is associated with a down-regulation of let-7b-5p, let-7c-5p, miR-27b-3p, and miR-10b-5p, which likely leads to a disinhibition of CXCR2 in the early postoperative period, being an important determinant of flow-induced angiogenesis in vivo. Other chemokines, like CCL3, CCL12, and CCL20 as well as CXCR4 and CCR5 were steadily overexpressed until POD 15, apparently acting as “intermediate-” and “late-phase” cytokines. Two chemokines, CXCL14 and CXCL21, were down-regulated most prominently in the intermediate and late postoperative periods. Both chemokines are potent inhibitors of angiogenesis in various types of cancers [38, 39]. In accordance with these studies, our data indicate that a down-regulation of these chemokines in AV shunts contributes to neoangiogenesis.

Interleukins were the second strongly over-expressed group of cytokines in our model, with IL1A and interleukin-1 beta (IL1B) showing a significant upregulation on POD 5. IL1A is known to promote angiogenesis by inducing inflammatory cell VEGF synthesis and secretion [40]. The expression of miR-27b-3p, which was twofold down-regulated on POD 5, negatively correlated with IL1A expression. A predicted target interaction was revealed for this miRNA/mRNA pair, indicating that up-regulation of IL1A in AV shunts is likely due to reduced post-transcriptional silencing by miR-27b-3p. IL1A overexpression in ECs of AV shunts was confirmed by immunohistochemical staining at the protein level for all AV shunt groups, whereas mRNA overexpression missed statistical significance on POD 10 (FC: 11, adjusted P: 0.12) and POD15 (FC: 7, adjusted P: 0.06) which is likely influenced by sample size.

IL-33, however, was down-regulated in all groups which is in accordance with the literature describing it as a marker for endothelial quiescence [41].

Another entity of proangiogenic cytokines overexpressed in AV shunts are members of the tumor necrosis factor (TNF) family, among these TNF alpha and its receptor (TNFR2) which were up-regulated in the early postoperative period. In cell cultures it has been shown that TNF is up-regulated in response to mechanical strain [42]. In accordance with our observation, Sainson et al. reported that a 2–3 day TNF pulse on endothelial cells stimulates angiogenesis by inducing platelet derived growth factor B (PDGFB) and vascular endothelial growth factor receptor 2 (VEGFR2) [43]. Lipopolysaccharide induced TNF factor (LITAF), a transcription factor which was continuously overexpressed in samples from AV shunts, upregulates TNF alpha as well as VEGF [44, 45]. Our data demonstrate that members of the TNF family are up-regulated in response to elevated vascular shear stress under in vivo conditions and are likely important determinants of flow-stimulated angiogenesis.

The sudden exposure of ECs to an arterial environment with elevated vascular shear stress leads to a state of EC activation, characterized by an upregulation of inflammatory factors such as chemokines and other cytokines, growth factors, as well as a downregulation of protective factors such as KLF2 [46]. As the process of angiogenesis is closely related to inflammation, with many pro-inflammatory factors such as several chemokines also acting pro-angiogenic, the miRNA-mRNA interactions we present constitute intriguing therapeutic targets for investigation in future studies pursuing treatment strategies for systemic inflammatory disorder such as rheumatoid arthritis.

### Oxygenation-associated genes

Our analysis revealed heme oxygenase-1 (HMOX1) to be among the most strongly up-regulated genes, with a peak expression on POD 5 (24-fold) and over tenfold up-regulation in AV shunts compared to controls throughout. HIF1A was significantly up-regulated on POD 5 and 10, however to a lesser extent compared to HMOX1. The oxygenation status of tissue is a major determinant of angiogenesis, and hypoxia is looked upon as the common denominator for angiogenesis and inflammation [47]. Oxygenation-sensitive proteins, namely HIF1A and HMOX1, respond to insufficient oxygen supply by inducing angiogenic growth factors of the VEGF/PDGF group and their receptors in strong synergy with inflammatory cytokines [48, 49]. HIF1A is known to be a key transcriptional activator of angiogenesis, transferring hypoxic stress into angiogenic stimuli through up-regulation of growth factors and induction of macrophage-mediated inflammation [27]. Bhang et al. demonstrated that combined HIF1A/HMOX1 gene therapy in mice is more efficient in inducing angiogenesis than either single-gene therapy [50]. In a mouse model of vascular injury, Kang et al. showed that HMOX1 is released from endothelial cells in response to vascular shear stress [51]. Therefore, our data indicate that HMOX1 likely represents an autonomous agent in flow-stimulated angiogenesis not requiring activation through cytokines. A strong down-regulation of NDRG2 was present in all groups of AV shunts. The *N*-myc oncogene (MYCN) was overexpressed on POD 5 and has been shown to repress NDRG2 [52]. Transfection of breast carcinoma cells with NDRG2 decreases the expression and proangiogenic activity of HIF1A and VEGF [53]. Hence, it is likely that the down-regulation of NDRG2 in AV shunts supports the proangiogenic effects of hypoxia-related genes.

S100A8 and S100A9, which code for calcium-binding proteins forming a heterodimer, calprotectin [54], showed a strong over-expression in AV shunts on POD 5. They were shown to promote endothelial tube formation in HUVEC cultures [55]. Ahn et al. demonstrated that transcriptional activation of HIF1A promotes angiogenesis through VEGF and S100A8 [56], illustrating the cooperative action of hypoxia-associated factors, S100A proteins, and VEGF. The main calprotectin receptor is toll-like receptor 4 (TLR4), which we found overexpressed on POD 5 and 15 as well [57], along with the proangiogenic toll-like receptor 6 (TLR6) [58]. We therefore assume that shear stress-induced HIF1A up-regulation in AV shunts, which is fostered by NDRG2 down-regulation, leads to concurrent over-expression of S100A8 and VEGF, promoting endothelial tube formation.

### Forkhead box C1

The transcription factor forkhead box C1 (FOXC1) was steadily down-regulated in AV shunts, most prominently on POD 15. We noted the expression level of miR-511-3p, which was strongly up-regulated on POD 15, to negatively correlate with FOXC1 expression. FOXC1 was found to be a predicted target of miR511-3p. FOXC1 is a key regulator of early angiogenesis, and determines endothelial cell fate and gene expression at early stages [59]. One of its physiological functions is the limitation of angiogenesis in the cornea, as demonstrated in FOXC1 knockout mice [59]. Conversely, conditional deletion of FOXC1 from VSMCs causes endothelial cell hyperplasia [60]. It has been demonstrated that FOXC1 deficiency leads to an up-regulation of matrix metalloproteinases (MMPs), including MMP9 [59, 61], which was also up-regulated on POD 5 in our study. MMP9 has the ability to cleave VEGF from the ECM, thereby increasing its concentration and facilitating angiogenesis [62]. Data from our study indicate that an up-regulation of miR511-3p, purportedly due to elevated shear stress in the AV shunts, leads to a down-regulation of FOXC1 which contributes to angiogenesis in rat AV shunts.

### Angiotensin and thrombospondins

We found both angiotensinogen (AGT) and angiotensin-converting enzyme (ACE) to be down-regulated in all groups, which is in accordance with the anti-angiogenic function of angiotensin and its cleaved derivatives [63]. The down-regulation of the angiotensin pathway may be driven by cytokines, as administration of TNF-alpha and IL-1 beta to EC cell cultures downregulates ACE [64]. Thrombospondins (THBS) are a group of ECM proteins associated with an anti-angiogenic function, in particular THBS1 and THBS2 [65], whereas THBS3 and THBS4 are poorly characterized so far [66]. We found both THBS3 and THBS4 to be strongly down-regulated in all groups, unlike THBS1 and THBS2. We ascertained this finding of the microarray analysis by RT-qPCR validation of THBS3. Thus, THBS3 and THBS4 may have as yet undescribed anti-angiogenic functions.

### Synaptojanin-2 binding protein/delta-like 1/apelin

In all AV shunt groups, a steady down-regulation of SYNJ2B was evident. SYNJ2BP expression correlated negatively with miR-223-3p—one of the most overexpressed miRNAs in AV shunts—for which predicted target interactions are present. Negative correlations were also present between SYNJ2BP and miR-19b-3p (POD 5 and 10) as well as miR-449a-5p (POD 15) which also have predicted binding sites on the SYNJ2BP-mRNA. To date, regulation of SYNJ2B expression by miRNAs has not yet been reported. SYN2JBP has only recently been recognized to be a regulator of angiogenesis. Adam et al. found that SYNJ2BP inhibits sprouting angiogenesis through enhancing the stability of the Notch ligands Delta-like 1 and 4 (DLL1, DLL4) and decreasing the expression of VEGF receptors [67]. Moreover, they demonstrated that SYNJ2BP-silenced human endothelial cells form a vascular network with increased vascular density in immunocompromised mice. Along with SYNJ2BP, we found DLL1 to be steadily down-regulated as well, while DLL4 expression was not significantly altered. DLL1 over-expression has been shown to attenuate tumor vascularization [68]. A crucial function of SYNJ2BP is the inhibition of apelin (APLN), a gene required for tip cell formation in sprouting blood vessels [67, 69]. Apelin was strongly overexpressed in all AV shunt groups, indicating its importance for endothelial tip formation in the developing vasculature of AV shunts. We assume that down-regulation of SYNJ2BP is mediated by post-transcriptional silencing through miR-223-3p and miR-19b-3p. Since a strong up-regulation of these two miRNAs was observed in all AV shunt groups, this phenomenon is likely triggered by hemodynamic forces due to elevated blood flow in the AV shunt. Disinhibition of apelin caused by SYNJBP down-regulation likely constitutes a previously undescribed early factor in flow-stimulated angiogenesis.

### Ephrin receptor kinases

The ephrin receptor kinases 3 and 4 (EPHA3, EPHA4) were significantly down-regulated in all AV shunt groups. EPHA2 was down-regulated only on POD 5 and showed a significant negative correlation with miR-130b-3p and miR-223-3p, for which predicted target interactions are present. The role of EPHA kinases in the formation and function of blood vessels is complex and only partially understood. It ranges from regulation of vascular permeability [70] to induction of angiogenesis [71]. Reports on the effects of EPHA2 inactivation are contradictory: Dobrzanski et al. described that interfering with EPHA2 signaling inhibits angiogenesis [72], whereas Okazaki et al. observed abundant endothelial sprouts and thick capillary diameters in EPHA2-deficient mice [73]. Our data support the notion that flow-induced upregulation of miR-130b-3p and miR-223-3p lead to post-transcriptional silencing of EPHA2. The exact role of EPHA2 in flow-stimulated angiogenesis, however, remains to be determined and warrants future studies.

### KLF2 and eNOS

With regard to the expression of KLF2 and eNOS in our model, our data confirm previous microarray analyses in carotid artery ligation models showing a downregulation of both genes due to a disturbed blood flow [74, 75]. The expression of both KLF2 and eNOS have been well characterized in response to various hemodynamic patterns of blood flow and existing data have shown that laminar flow patterns lead to an activation of both genes [76], whereas a disturbed blood flow leads to a downregulation of eNOS and KLF2 as has been demonstrated in carotid artery ligation models [74, 75]. To date, systematic investigations of blood flow in the AV shunt model are limited to assessment of absolute levels of blood flow and shear stress over time, which have been performed by our group and others [21, 77] but the exact flow pattern have not yet been investigated. Our data on KLF2 and eNOS expression fit with a pattern of disturbed flow, however, systematic analysis of flow patterns are needed to better define their impact on gene expression in this model. Since it is well known that eNOS mediates pro-angiogenic effects, our results indicate that the eNOS pathway is not a main determinant of neoangiogenesis in the AV shunt model.

### Vascular growth factors

Growth factors of the VEGF and PDGF families and their receptors are core inducers of angiogenesis [78, 79]. In our study VEGFA and PDGFA were overexpressed on POD 5 and 10, and PDGFB was overexpressed on POD 15. Moreover, the downstream effector of VEGF signaling, phospholipase C gamma 2 (PLCG2) is up-regulated on POD 5, fitting with its role as an early determinant of sheer stress-induced signaling, as determined by Bazmara et al. through in silico analysis [80]. All aforementioned pathways, up-regulation of cytokines, oxygenation-associated genes, as well as down-regulation of the FOXC1 and SYNJ2BP, culminate in an overexpression of vascular growth factors. The CXCL2/CXCR2 pathway, which we found strongly up-regulated in AV shunts, activates PLC beta 2 and PLCG2 as well as phosphoinositide-3-kinase (PI3K) and its downstream gene HIF1A, all of which act as VEGF effectors [31].

We demonstrate a specific profile of deregulated miRNAs in vascular tissue from a rat model of elevated vascular shear stress and indicate significant correlations and predicted interactions of these miRNAs with genes that regulate angiogenesis. Our study extends the current understanding of miRNA-regulated pathways in flow-stimulated angiogenesis and identifies several promising targets for future studies on RNA-based therapeutic interventions [81]. Future translational research in this field may deliver new therapeutic approaches for miRNA-driven enhancement of local angiogenesis, aimed at either rescuing critically ischemic tissue e.g. in patients with diabetic angiopathy or myocardial ischemia, or at fostering the engraftment of tissue transplants. The data presented here may also be applicable in future translational studies in the field of tissue engineering. The AV loop model is a reliable tissue engineering technique for creation of vascularized and transplantable soft tissue units and has a great potential for successful translation from animal models into clinical trials in the future [82]. The miRNA profile and specific candidate genes we report, can serve as targets for selective enhancement of neovascularization using synthetic miRNA-mimics or antagomirs. Conversely, the same understanding may provide new clues for the suppression of angiogenesis in cancer.

## Conclusions

Our in vivo data provide evidence that flow-stimulated angiogenesis resulting from elevated vascular shear stress is driven by an up-regulation of cytokines (e.g. CXCL2, IL1A) as well as oxygenation-associated genes (HIF1A, HMOX1), and by a down-regulation of the embryonic transcription factor FOXC1 as well as the mitochondrial membrane protein SYNJ2BP. Significant inverse correlations of the expression levels of these genes with their interacting miRNAs, namely the up-regulated miR-223-3p, miR-130b-3p, miR-19b-3p, miR-449a-5p, and miR-511-3p as well as the down-regulated miR-27b-3p, miR-10b-5p, let-7b-5p, and let-7c-5p, illustrate that a deregulation of miRNA expression is likely responsible for the observed gene and protein expression changes. Thus, the timeline of events in flow-stimulated angiogenesis in vivo appears to start with elevated vascular shear stress leading to a deregulation of specific miRNAs, which affect the expression of CXCR2, IL1A, FOXC1, SYNJ2BP, and EPHA2. Elevated expression of chemokines, interleukins, as well as of HIF1A and HMOX1 along with a down-regulation of SYNJ2BP and FOXC1 promotes VEGF expression and neoangiogenesis.

## Additional file

**Additional file 1.** Additional tables.

## Abbreviations

ACE: angiotensinogen converting enzyme
AGT: angiotensinogen
AP2: adaptor protein 2
AV shunt: arteriovenous shunt
CCL: C–C motif chemokine ligand
CXCL: C–X–C motif chemokine ligand
CXCR: C–X–C motif chemokine receptor
CT: computed tomography
DLL: delta-like
EC: endothelial cell
ECM: extracellular matrix
EPHA2: ephrin receptor kinase 2
eNOS: endothelial nitric oxide synthase
FC: fold-change
FOXC1: forkhead box C1
GAPDH: glyceraldehyde 3-phosphate dehydrogenase
GO: gene ontology
HIF1A: hypoxia-inducible factor 1-alpha
HMOX1: heme oxygenase 1
HPRT: hypoxanthine–guanine phosphoribosyltransferase
HUVEC: human umbilical vein endothelial cell
IL1A: interleukin-1 alpha
KLF: kruppel-like factor
LITAF: lipopolysaccharide induced TNF factor
MMP: matrix metalloproteinase
miRNA: microRNA
mRNA: messenger RNA
NDRG2: *N*-myc downstream regulated gene 2
NRT: no reverse transcriptase control
NTC: no template control
PBS: phosphate-buffered saline
PDGF: platelet derived growth factor
PFA: paraformaldehyde
PI3K: phosphoinositide-3-kinase
PLC: phospholipase C
POD: postoperative day
RNA: ribonucleic acid
ROI: region of interest
RT-PCR: reverse transcription polymerase chain reaction
RT-qPCR: quantitative real-time polymerase chain reaction
SYNJ2BP: synaptojanin-2 binding protein
THBS: thrombospondin
TLR: toll-like receptor
TNF: tumor necrosis factor
TNFR: tumor necrosis factor receptor
VEGF: vascular endothelial growth factor
VSMC: vascular smooth muscle cell
