# Supplementary material for: MicroRNA-regulated pathways of flow-stimulated angiogenesis and vascular remodeling in vivo
Source: J Transl Med. 2019 Jan 11;17:22. doi: 10.1186/s12967-019-1767-9 (PMC6330440; doi:10.1186/s12967-019-1767-9)
Supplement: Supplementary file 1 — Additional file 1. Additional tables. [file 12967_2019_1767_MOESM1_ESM.doc]

**MicroRNA-Regulated Pathways of Flow-Stimulated Angiogenesis and Vascular Remodeling *in vivo***

Dominic Henn, MD*^1^, Masood Abu-Halima, PhD*^2^ Dominik Wermke, MS^3^, Florian Falkner, BS^1^, Benjamin Thomas, MD^1^, Christoph Köpple, MD^1^, Nicole Ludwig, PhD^2^, Matthias Schulte, PhD^1^, Marc A. Brockmann, MD^4^, Yoo-Jin Kim, MD^5^, Justin M. Sacks, MD, MBA^6^, Ulrich Kneser, MD^1^, Andreas Keller, PhD^3^, Eckart Meese, PhD^+2^, Volker J. Schmidt, MD^+1^

* and + The authors contributed equally to this work.

**Table S1: Employed Gene Ontology (GO) terms for GO enrichment analysis**

| **Go Term** | **Description** |
| --- | --- |
| **Positive Regulation of Angiogenesis** | |
| GO:1903672 | positive regulation of sprouting angiogenesis |
| GO:0045766 | positive regulation of angiogenesis |
| GO:0090050 | positive regulation of cell migration involved in sprouting angiogenesis |
| **Negative Regulation of Angiogenesis** | |
| GO:1903671 | negative regulation of sprouting angiogenesis |
| GO:1903588 | negative regulation of blood vessel endothelial cell proliferation involved in sprouting angiogenesis |
| GO:0090051 | negative regulation of cell migration involved in sprouting angiogenesis |
| GO:0016525 | negative regulation of angiogenesis |
| GO:1903588 | negative regulation of blood vessel endothelial cell proliferation involved in sprouting angiogenesis |

**Table S2: Differentially expressed micro-RNAs (miRNAs) in arteriovenous (AV) shunts compared to controls in miRNA microarray analysis. p ≤ 0.05 for all comparisons.**

| **A) POD5 compared to controls** | | | | | | | |
| --- | --- | --- | --- | --- | --- | --- | --- |
| **miRNA** | **Mean POD5** | **Mean Control** | **Log Mean** | **Fold-Change** | **Regulation** | ***P-*value** | **Adjusted *P-*value** |
| miR-143-3p | 7.99 | 10.74 | -2.75 | -6.71 | Down | 6.25x10^-6^ | 0.0014 |
| miR-195-5p | 5.90 | 8.15 | -2.25 | -4.77 | Down | 2.79x10^-5^ | 0.0029 |
| miR-497-5p | 5.63 | 7.76 | -2.13 | -4.37 | Down | 9.27x10^-6^ | 0.0014 |
| miR-370-3p | 3.74 | 5.85 | -2.11 | -4.32 | Down | 0.0004 | 0.0181 |
| rno-let-7c-5p | 10.29 | 12.40 | -2.10 | -4.30 | Down | 0.0001 | 0.0087 |
| miR-10b-5p | 5.84 | 7.75 | -1.91 | -3.76 | Down | 0.0006 | 0.0127 |
| miR-99a-5p | 6.37 | 8.28 | -1.91 | -3.76 | Down | 0.0002 | 0.0129 |
| miR-196a-5p | 4.23 | 6.02 | -1.79 | -3.46 | Down | 0.0003 | 0.0147 |
| rno-let-7e-5p | 7.01 | 8.74 | -1.73 | -3.33 | Down | 0.0008 | 0.0244 |
| miR-196b-5p | 4.76 | 6.48 | -1.72 | -3.30 | Down | 0.0003 | 0.0015 |
| rno-let-7b-5p | 9.99 | 11.68 | -1.69 | -3.23 | Down | 0.0004 | 0.0181 |
| miR-23b-3p | 9.15 | 10.68 | -1.53 | -2.88 | Down | 0.0001 | 0.0107 |
| miR-10a-5p | 5.95 | 7.47 | -1.52 | -2.87 | Down | 0.0008 | 0.0243 |
| miR-27b-3p | 7.99 | 9.46 | -1.47 | -2.77 | Down | 0.0001 | 0.0107 |
| miR-204-5p | 3.78 | 5.24 | -1.46 | -2.75 | Down | 0.0002 | 0.0137 |
| miR-125a-5p | 6.17 | 7.60 | -1.43 | -2.69 | Down | 0.0006 | 0.0217 |
| miR-143-5p | 2.38 | 3.40 | -1.01 | -2.02 | Down | 0.0004 | 0.0181 |
| miR-28-3p | 1.13 | 1.95 | -0.81 | -1.75 | Down | 0.0012 | 0.0490 |
| miR-3574 | 1.62 | 2.35 | -0.74 | -1.67 | Down | 0.0017 | 0.0280 |
| miR-362-3p | 4.39 | 3.57 | 0.82 | 1.76 | Up | 0.0009 | 0.0051 |
| miR-363-3p | 3.79 | 2.51 | 1.28 | 2.43 | Up | 5.39x10^-5^ | 0.0182 |
| miR-15b-5p | 10.66 | 9.25 | 1.41 | 2.66 | Up | 8.64x10^-6^ | 0.0014 |
| miR-7a-5p | 4.74 | 3.32 | 1.42 | 2.67 | Up | 0.0002 | 0.0129 |
| miR-20a-5p | 9.05 | 7.43 | 1.62 | 3.07 | Up | 0.0001 | 0.0087 |
| miR-188-5p | 8.25 | 6.55 | 1.70 | 3.25 | Up | 0.0008 | 0.0243 |
| miR-301a-3p | 6.29 | 4.58 | 1.71 | 3.27 | Up | 0.0021 | 0.0317 |
| miR-340-5p | 4.45 | 2.72 | 1.73 | 3.32 | Up | 9.43x10^-5^ | 0.0408 |
| miR-19b-3p | 9.47 | 7.61 | 1.86 | 3.63 | Up | 1.18-10^-5^ | 0.0014 |
| miR-142-3p | 9.74 | 7.73 | 2.01 | 4.03 | Up | 4.27x10^-6^ | 0.0014 |
| miR-32-3p | 6.14 | 4.07 | 2.07 | 4.21 | Up | 0.0012 | 0.0079 |
| miR-130b-3p | 5.28 | 3.12 | 2.16 | 4.46 | Up | 0.0016 | 0.0400 |
| miR-19a-3p | 7.42 | 5.01 | 2.41 | 5.32 | Up | 0.0008 | 0.0243 |
| miR-142-5p | 7.30 | 4.77 | 2.53 | 5.78 | Up | 0.0012 | 0.0317 |
| miR-223-3p | 11.34 | 8.59 | 2.76 | 6.75 | Up | 8.84x10^-9^ | 6.70x10^-6^ |
|  |  |  |  |  |  |  |  |
| **B) POD10 compared to controls** | | | | | | | |
| **miRNA** | **Mean POD10** | **Mean Control** | **Log Mean** | **Fold Change** | **Regulation** | ***P-*value** | **Adjusted *P-*value** |
| miR-203a-3p | 1.95 | 2.56 | -0.61 | -1.53 | Down | 6.71x10^-5^ | 0.0097 |
| miR-33-5p | 2.47 | 1.93 | 0.53 | 1.45 | Up | 0.0008 | 0.0409 |
| miR-34a-3p | 2.97 | 2.23 | 0.74 | 1.67 | Up | 3.98x10^-7^ | 0.0003 |
| miR-222-5p | 2.87 | 2.08 | 0.78 | 1.72 | Up | 3.47x10^-5^ | 0.0097 |
| miR-27a-3p | 10.23 | 9.41 | 0.81 | 1.76 | Up | 0.0001 | 0.0108 |
| miR-674-3p | 3.75 | 2.81 | 0.94 | 1.91 | Up | 0.0004 | 0.0238 |
| miR-19b-3p | 8.65 | 7.61 | 1.04 | 2.06 | Up | 0.0007 | 0.0364 |
| miR-34b-5p | 4.22 | 3.17 | 1.05 | 2.07 | Up | 0.0001 | 0.0108 |
| miR-34c-5p | 4.12 | 2.88 | 1.23 | 2.35 | Up | 0.0002 | 0.0133 |
| miR-340-5p | 4.21 | 2.72 | 1.49 | 2.82 | Up | 0.0003 | 0.0193 |
| miR-221-3p | 7.48 | 5.95 | 1.53 | 2.88 | Up | 0.0003 | 0.0226 |
| miR-542-3p | 4.27 | 2.73 | 1.53 | 2.89 | Up | 0.0010 | 0.0487 |
| miR-223-3p | 10.57 | 8.59 | 1.98 | 3.95 | Up | 0.0005 | 0.0266 |
| miR-34a-5p | 9.09 | 6.44 | 2.66 | 6.30 | Up | 7.66x10^-5^ | 0.0097 |
| miR-31a-3p | 5.82 | 3.05 | 2.78 | 6.86 | Up | 5.13x10^-5^ | 0.0097 |
| miR-31a-5p | 8.49 | 4.29 | 4.21 | 18.49 | Up | 5.35x10^-5^ | 0.0097 |
|  |  |  |  |  |  |  |  |
| **C) POD15 compared to controls** | | | | | | | |
| **miRNA** | **Mean POD15** | **Mean Control** | **Log Mean** | **Fold Change** | **Regulation** | ***P-*value** | **Adjusted *P-*value** |
| miR-1b | 0.71 | 5.35 | -4.65 | -25.04 | Down | 0.0010 | 0.0248 |
| miR-486 | 4.52 | 8.81 | -4.30 | -19.64 | Down | 8.94x10^-5^ | 0.0046 |
| miR-451-5p | 8.18 | 11.32 | -3.13 | -8.78 | Down | 0.0010 | 0.0243 |
| miR-144-3p | 4.04 | 6.65 | -2.61 | -6.09 | Down | 0.0024 | 0.0370 |
| miR-144-5p | 2.82 | 4.71 | -1.89 | -3.70 | Down | 0.0026 | 0.0370 |
| miR-10b-5p | 6.65 | 7.85 | -1.21 | -2.31 | Down | 0.0025 | 0.0370 |
| miR-3593-3p | 4.88 | 6.14 | -1.26 | -2.40 | Down | 0.0022 | 0.0354 |
| rno-let-7b-5p | 10.53 | 11.78 | -1.25 | -2.39 | Down | 0.0038 | 0.0458 |
| miR-10a-5p | 6.38 | 7.57 | -1.18 | -2.27 | Down | 0.0002 | 0.0080 |
| miR-204-5p | 3.84 | 5.33 | -1.50 | -2.82 | Down | 0.0014 | 0.0277 |
| miR-205 | 1.93 | 3.03 | -1.10 | -2.14 | Down | 0.0020 | 0.0345 |
| miR-23b-3p | 9.80 | 10.78 | -0.98 | -1.97 | Down | 0.0008 | 0.0225 |
| miR-582-3p | 3.12 | 3.97 | -0.86 | -1.81 | Down | 0.0026 | 0.0370 |
| miR-193b-3p | 3.19 | 3.94 | -0.74 | -1.67 | Down | 9.68x10^-5^ | 0.0046 |
| miR-29c-5p | 3.12 | 3.90 | -0.77 | -1.71 | Down | 0.0027 | 0.0370 |
| miR-99a-3p | 2.86 | 3.53 | -0.66 | -1.58 | Down | 0.0009 | 0.0233 |
| miR-449a-5p | 3.41 | 2.60 | 0.81 | 1.75 | Up | 0.0013 | 0.0273 |
| miR-3075 | 3.15 | 2.39 | 0.76 | 1.69 | Up | 0.0035 | 0.0448 |
| miR-33-5p | 2.67 | 2.00 | 0.67 | 1.59 | Up | 0.0025 | 0.0370 |
| miR-450b-5p | 3.06 | 2.38 | 0.68 | 1.60 | Up | 0.0001 | 0.0055 |
| miR-204-3p | 2.04 | 1.43 | 0.61 | 1.53 | Up | 0.0001 | 0.0050 |
| miR-22-3p | 10.34 | 9.27 | 1.07 | 2.10 | Up | 0.0027 | 0.0370 |
| miR-6326 | 3.45 | 2.60 | 0.85 | 1.81 | Up | 0.0011 | 0.0252 |
| miR-7a-5p | 4.31 | 3.40 | 0.91 | 1.87 | Up | 0.0010 | 0.0248 |
| miR-363-3p | 3.43 | 2.57 | 0.86 | 1.81 | Up | 0.0018 | 0.0332 |
| miR-218a-5p | 5.02 | 3.91 | 1.11 | 2.16 | Up | 0.0022 | 0.0355 |
| miR-542-5p | 4.46 | 3.14 | 1.32 | 2.50 | Up | 0.0016 | 0.0296 |
| miR-19b-3p | 8.64 | 7.71 | 0.93 | 1.90 | Up | 0.0039 | 0.0464 |
| miR-27a-3p | 10.60 | 9.52 | 1.09 | 2.12 | Up | 1.13x10^-5^ | 0.0012 |
| miR-34a-3p | 3.32 | 2.29 | 1.02 | 2.03 | Up | 1.05x10^-7^ | 7.95x10^-5^ |
| miR-222-5p | 3.24 | 2.15 | 1.09 | 2.13 | Up | 0.0001 | 0.0050 |
| miR-674-3p | 3.99 | 2.89 | 1.10 | 2.14 | Up | 1.69x10^-5^ | 0.0016 |
| miR-181b-1-3p | 2.23 | 1.22 | 1.02 | 2.02 | Up | 0.0002 | 0.0056 |
| miR-181a-1-3p | 4.18 | 3.07 | 1.10 | 2.15 | Up | 1.54x10^-6^ | 0.0003 |
| miR-362-3p | 4.64 | 3.65 | 0.99 | 1.98 | Up | 0.0013 | 0.0273 |
| miR-450a-5p | 5.33 | 3.98 | 1.35 | 2.56 | Up | 9.30x10^-5^ | 0.0046 |
| miR-582-5p | 4.28 | 3.12 | 1.16 | 2.23 | Up | 1.92x10^-5^ | 0.0016 |
| miR-455-3p | 5.08 | 4.03 | 1.04 | 2.06 | Up | 0.0030 | 0.0389 |
| miR-16-5p | 11.80 | 10.74 | 1.06 | 2.09 | Up | 0.0008 | 0.0225 |
| miR-221-3p | 7.89 | 6.04 | 1.84 | 3.58 | Up | 0.0001 | 0.0050 |
| miR-322-5p | 8.27 | 6.57 | 1.70 | 3.25 | Up | 4.89x10^-5^ | 0.0034 |
| miR-340-5p | 4.69 | 2.79 | 1.90 | 3.74 | Up | 0.0003 | 0.0089 |
| miR-34b-5p | 4.69 | 3.24 | 1.45 | 2.73 | Up | 5.28x10^-6^ | 0.0007 |
| miR-34c-5p | 4.67 | 2.95 | 1.71 | 3.27 | Up | 2.08x10^-6^ | 0.0003 |
| miR-455-5p | 4.91 | 3.34 | 1.57 | 2.96 | Up | 4.35x10^-7^ | 0.0001 |
| miR-542-3p | 4.64 | 2.81 | 1.84 | 3.57 | Up | 3.74x10^-5^ | 0.0028 |
| miR-210-3p | 7.11 | 4.57 | 2.55 | 5.85 | Up | 0.0036 | 0.0450 |
| miR-146b-5p | 8.26 | 6.07 | 2.19 | 4.56 | Up | 0.0023 | 0.0355 |
| miR-223-3p | 11.04 | 8.69 | 2.36 | 5.12 | Up | 0.0009 | 0.0236 |
| miR-34a-5p | 9.45 | 6.53 | 2.91 | 7.53 | Up | 7.50x10^-5^ | 0.0046 |
| miR-511-3p | 5.89 | 3.18 | 2.70 | 6.52 | Up | 0.0004 | 0.0114 |
| miR-146a-5p | 9.87 | 6.57 | 3.30 | 9.84 | Up | 0.0016 | 0.0296 |
| miR-31a-3p | 6.36 | 3.12 | 3.24 | 9.48 | Up | 5.02x10^-7^ | 0.0001 |
| miR-31a-5p | 8.83 | 4.37 | 4.46 | 22.08 | Up | 9.36x10^-5^ | 0.0046 |

**Table S3: Selected mRNAs with differential expression in the microarray analysis between AV shunts and controls. POD = postoperative day. p ≤ 0.05 for all comparisons where a fold-change is given. NS = not significant.**

| **Gene Symbol** | **Gene Name** | **Fold Change** | | |
| --- | --- | --- | --- | --- |
|  |  | **POD 5** | **POD 10** | **POD 15** |
| **Cytokines** | | | | |
| AP2S1 | Adaptor related protein complex 2 sigma 1 subunit | 2.46 | 1.63 | NS |
| CCL12 | C-C motif chemokine ligand 12 | 34.78 | 11.70 | 15.34 |
| CCL20 | C-C motif chemokine ligand 20 | 14.82 | 4.19 | 5.74 |
| CCL21 | C-C motif chemokine ligand 21 | -7.33 | -24.65 | -35.35 |
| CCL3 | C-C motif chemokine ligand 3 / macrophage inflammatory protein 1-alpha | 30.54 | 11.38 | 11.83 |
| CCL4 | C-C motif chemokine ligand 4 | 8.37 | NS | 6.44 |
| CCL6 | C-C motif chemokine ligand 6 | 5.14 | NS | NS |
| CCL9 | C-C motif chemokine ligand 9 | 7.56 | NS | NS |
| CCR1 | C-C motif chemokine receptor 1 | 11.24 | NS | NS |
| CCR2 | C-C motif chemokine receptor 2 | 2.48 | 1.40 | NS |
| CCR5 | C-C motif chemokine receptor 5 | 7.00 | 4.02 | 6.07 |
| CXCL11 | C-X-C motif chemokine ligand 11 | 1.78 | NS | NS |
| CXCL14 | C-X-C motif chemokine ligand 14 | -6.70 | -21.05 | -17.06 |
| CXCL2 | C-X-C motif chemokine ligand 2 | 42.39 | NS | NS |
| CXCL3 | C-X-C motif chemokine ligand 3 | 49.00 | NS | NS |
| CXCR2 | C-C motif chemokine receptor 2 | 10.92 | NS | NS |
| CXCR4 | C-X-C motif chemokine receptor 4 | 4.32 | 5.26 | 5.21 |
| IL1A | Interleukin 1 alpha | 35.66 | NS | NS |
| IL1B | Interleukin 1 beta | 12.65 | NS | NS |
| IL1R2 | Interleukin 1 receptor type 2 | 23.22 | 6.92 | 6.18 |
| IL1RL1 | Interleukin 1 receptor-like | 4.87 | 6.96 | 6.87 |
| IL1RN | Interleukin 1 receptor antagonist | 14.33 | 8.42 | 9.18 |
| IL33 | Interleukin 33 | -3.91 | -4.44 | -5.63 |
| LITAF | Lipopolysaccharide induced TNF factor | 3.73 | 2.53 | 2.61 |
| TNF | Tumor necrosis factor | 8.80 | 3.41 | NS |
| TNFR2 | Tumor necrosis factor receptor 2 | 6.38 | NS | 2.87 |
| TNFRSF5 (CD40) | Tumor necrosis factor receptor superfamily member 5 | 1.69 | NS | 2.08 |
| TRAF3 | Tumor necrosis factor receptor associated factor 3 | 1.57 | NS | NS |
| **VEGF / PDGF Signaling** | | | | |
| CSK (C-Src) | C-Src-tyrosine kinase | 1.71 | 1.75 | 1.94 |
| PDGFA | Platelet derived growth factor A | 2.16 | 2.41 | NS |
| PDGFB | Platelet derived growth factor B | NS | NS | 1.84 |
| PI3K | Phosphoinositide 3-kinase | 2.50 | 1.77 | 1.78 |
| PLCG2 | Phospholipase C gamma 2 | 1.89 | NS | NS |
| TRAF6 | Tumor necrosis factor receptor associated factor 6 | 1.51 | NS | NS |
| VEGFA | Vascular endothelial growth factor-A | 4.41 | 3.11 | NS |
| **Others** | | | | |
| ACE | Angiotensin-converting enzyme | -8.64 | -3.71 | -8.91 |
| AGT | Angiotensinogen | -5.77 | -5.75 | -4.99 |
| APLN | Apelin | 8.06 | 19.88 | 18.83 |
| DLL1 | Delta-like 1 | -5.18 | -2.19 | -1.99 |
| EPHA2 | Ephrin receptor A2 | -3.18 | NS | NS |
| EPHA3 | Ephrin receptor A3 | -3.77 | -4.05 | -2.89 |
| EPHA4 | Ephrin receptor A4 | -1.86 | -1.88 | -1.82 |
| FOXC1 | Forkhead box C1 | -5.55 | -4.25 | -6.29 |
| HIF1A | Hypoxia-inducible factor 1 alpha | 1.97 | 1.58 | NS |
| HMOX1 | Heme oxygenase 1 | 23.81 | 13.94 | 15.81 |
| KLF2 | Kruppel-like factor 2 | -2.25 | NS | NS |
| MMP13 | Matrix metalloproteinase 12 | 4.53 | NS | 11.06 |
| MMP7 | Matrix metalloproteinase 7 | 5.46 | NS | NS |
| MMP9 | Matrix metalloproteinase 9 | 4.17 | NS | NS |
| MYCN | N-myc oncogene | 2.86 | NS | NS |
| NDRG2 | N-myc downstream regulated gene 2 | -21.00 | -10.90 | -8.48 |
| NOS3 (eNOS) | Nitric oxide synthase 3 | NS | NS | -2.53 |
| S100A8 | S100 calcium-binding protein A8 | 13.69 | NS | 3.48 |
| S100A9 | S100 calcium-binding protein A9 | 15.56 | 5.83 | NS |
| SYNJ2BP | Synaptojanin 2-binding protein | -2.73 | -2.19 | -1.99 |
| THBS3 | Thrombospondin 3 | -8.78 | -5.79 | -6.38 |
| THBS4 | Thrombospondin 4 | -43.92 | -46.05 | -40.72 |
| TLR4 | Toll-like receptor 4 | 2.06 | NS | 1.76 |
| TLR6 | Toll-like receptor 6 | 4.10 | NS | 10.05 |
